# Catfish Epidermal Preparation Accelerates Healing of Damaged Nerve in a Sciatic Nerve Crush Injury Rat Model

**DOI:** 10.3389/fphar.2021.632028

**Published:** 2021-04-14

**Authors:** Waleed M. Renno, Mohammad Afzal, Bincy Paul, Divya Nair, Jijin Kumar, Jassim M. Al-Hassan

**Affiliations:** ^1^ Department of Anatomy, Faculty of Medicine, Kuwait University, Safat, Kuwait; ^2^ Biological Sciences, Faculty of Science, Kuwait University, Safat, Kuwait

**Keywords:** neuroprotection, neuroregeneration, nerve injury, neurobehavioral, catfish epidermal mucus extracts

## Abstract

Preliminary investigations showed that preparations from Arabian Gulf catfish (*Arius bilineatus*, Val) epidermal gel secretion (PCEGS) exhibit potent anti-inflammatory and healing properties as shown in our previous clinical trials for the healing of non-healing diabetic foot ulcers, chronic back pain, and some other neurological disorders. Here, we report for the first time a unique preparation containing only proteins and lipids (soluble protein fraction B, SPF-FB), derived from the PCEGS accelerated the healing and recovery of sensory-motor functions of experimental sciatic nerve crush injury in rats with its unique neuroprotective and neuroregenerative properties on the spinal neurons and peripheral nerve fibers. Male rats were randomly assigned to five groups: (I) NAÏVE, (II) SHAM, (III) CRUSH treated with saline, (IV) CRUSH + SPF-FB treated with 3 mg/kg intraperitoneally (IP) and (V) CRUSH + SPF-FB treated with 6 mg/kg subcutaneously (SC) groups. The crush groups III, IV and V underwent sciatic nerve crush injury, followed by treatment daily for 14 days with saline, SPF-FB IP and SPF-FB SC. All animals were tested for the neurobehavioral parameters throughout the 6 weeks of the study. Sciatic nerve and spinal cord tissues were processed for light and electron histological examinations, stereological analysis, immunohistochemical and biochemical examinations at Week 4 and Week 6 post-injury. Administration of SPF-FB IP or SC significantly enhanced the neurobehavioral sensory and motor performance and histomorphological neuroregeneration of the sciatic nerve-injured rats. The stereological evaluation of the axon area, average axon perimeters, and myelin thickness revealed significant histomorphological evidence of neuroregeneration in the FB-treated sciatic nerve crush injured groups compared to controls at 4 and 6 weeks. SPF-FB treatment significantly prevented the increased in NeuN-immunoreactive neurons, increased GFAP immunoreactive astrocytes, and decreased GAP-43. We conclude that SPF-FB treatment lessens neurobehavioral deficits, enhances axonal regeneration following nerve injury. We conclude that SPF-FB treatment lessens neurobehavioral deficits and enhances axonal regeneration following nerve injury, as well as protects spinal neurons and enhances subcellular recovery by increasing astrocytic activity and decreasing GAP-43 expression.

## Introduction

There are four morphologically distinct species of ariid catfish in the northern regions of the Arabian Gulf, including *Arius thalassinus, A. dussumieri, A. tenuispinis and A. bilineatus* (Al-Hassan et al., 1988). *Arius bilineatus*, Val., which is the most abundant species migrating to Kuwait territorial waters during the summer months, is the focus of this study. All our publications before 1988 referred to *Arius bilineatus* as *Arius thallassinus. A. bilineatus* was misnamed by fish taxonomists and corrected in 1988 (Al-Hassan et al., 1988). *A. bilineatus* secretes copious amounts of thick gel-like proteinaceous material through its epidermis when threatened or injured. This is separate from the more aqueous mucus secretion, which the fish elaborates from mucous cells after removing the proteinaceous material. The club cells in the epidermis appear to be the prime source of the proteinaceous secretions. The gel-like material comprises 85% proteins and 13.4% lipids with small amounts (1.6%) of carbohydrates and nucleic acid components. The epidermal secretions’ composition is entirely different from the mucus, which is mainly composed of polysaccharides and, therefore, must serve different functions from those served by mucus. We previously hypothesized that this epidermal secretion is for protecting the fish against injury (Al-Hassan et al., 1982, Al-Hassan et al., 1985; Al-Hassan et al., 1986a; Al-Hassan et al., 1987a).

Our previous studies have shown that preparations from Arabian Gulf catfish (*Arius bilineatus*, Val) epidermal gel secretion (PCEGS) are composed of a mixture of biochemically and pharmacologically active lipids and proteins. These include components that act on blood, such as a hemolytic enzyme (Al-Lahham et al., 1987), a plasma clotting factor that converts factor X to Xa (Summers et al., 1985), and a hemagglutination factor (Al-Hassan et al., 1986b). Other components found are vasoconstricting agents Al-Hassan et al., 1986c; Thulesius et al., 1983; Al-Bow et al., 1997) prostaglandins (Al-Hassan et al., 1986a; Al-Hassan et al., 1987b), such as PGF_2α_, thromboxane B_2_, 6-keto prostaglandin F_1α_, 13,14-dihydroxy, 15-keto prostaglandin F_2α_, 8-epi prostaglandin F_2α_ (Al-Hassan et al. 1998), high levels of platelet-activating factors (PAF) (Plattner et al., 1988; Summers et al., 1991), arachidonic acid (Al-Hassan et al., 1986b; Al-Hassan et al., 1987b) and 14 steroids (Al-Hassan et al., 2016a). It also includes a factor that activates phospholipase A_2_ (Al-Hassan et al., 1987b).

Recently we have shown that different catfish skin lipid-soluble fractions have anti-proliferative and anti-inflammatory activities against pancreatic, lung, prostate, skin, liver, and breast cancer cell lines (Yang et al., 2016; Al-Hassan et al., 2019). Further, our coworkers have shown that the lipid fraction of PCEGS, C-20 fatty acid (12,15-epoxy-13,14-dimethyleicosa-12,14-dienoic acid, a furanoic acid or F-6) vigorously induces neutrophil extracellular trap formation (Khan et al., 2019). The PCEGS lipid fraction (Ft-3) dose-dependently kills two cancer cell lines (breast MDA MB-231 and leukemic K-562) (Al-Hassan et al., 2019). Also, SPF-FB exhibited acceleration of wound healing (Pace-Asciak et al., 2017; Al-Hassan et al., 1991; Al-Hassan et al., 2020) potent anti-inflammatory and therapeutic activities, as shown by our previous clinical trials for the healing of non-healing diabetic foot ulcers (Al-Hassan 1990), chronic back pain, and other neurological disorders (Al-Hassan et al., 2005; Al-Hassan et al., 2006; Al-Hassan et al., 2007). These preliminary data have indicated that SPF-FB preparations had a noticeable effect on neuropathy in the diabetic foot and potent therapeutic effects on chronic back and joint pain (Al-Hassan, 1990; Al-Hassan et al., 2005; Al-Hassan et al., 2006; Al-Hassan et al., 2007). In separate medical applications for treating some neurological problems, a fraction of PCEGS [fraction B of the soluble protein fraction (SPF-FB)] was used topically to treat other neurological problems. Our obtained preliminary results with one paraplegia case (not a polio case) paralyzed from the waist downward for 18 years, one chronic demyelinated polyneuropathy case, and six cases of cervical problems that threatened quadriplegia showed complete recovery, while one case of quadriplegia showed noticeable improvement (Al-Hassan, 1990; Al-Hassan et al., 2005; Al-Hassan et al., 2006; Al-Hassan et al., 2007). These remarkable results warranted detailed specialized research to explain the mode and mechanism of action of Fraction B on the nervous system. These results led us to study in detail the effects of SPF-FB on sciatic nerve crush injury, as this involves most of the symptoms shown in the treated cases. It is hoped that the results of this study might lead to a new line of treatments for some neurological problems that have been slow to heal or even impossible to treat with conventional treatments.

Sciatic nerve crush injury has been widely accepted as the best model to study peripheral nerve regeneration (Thalhammer et al., 1995; Luis et al., 2007). It allows investigators to perform a standard direct trauma in rats and results in a lesion similar to those seen in patients with peripheral nerve injury (Hussain et al., 2018; Renno et al., 2012, Renno et al., 2013, Renno et al., 2016, Renno et al., 2017). The nerve crush model also represents an excellent model to examine mild forms of nerve compression injury or neuropathy that are known to be associated with myelin-related neurodegenerative diseases, where nerve function deteriorates as these severe chronic disorders progress.

In this study, our hypothesis utilized the therapeutic and regenerative properties of the potent wound and diabetic ulcer healing preparation, Fraction B, for healing of damaged nerves. The biologically and pharmacologically active protein and lipids extracts from the catfish skin work in synergism and possess neuroprotective and neuroregenerative properties to modify, regulate and modulate the incapacitated peripheral nerve microenvironment in the direction of rather more accommodating than inhibitory effect, which in turn may increase the way to axonal. The novel aspect of this study is that it investigates for the first time the effects of fraction B on the neuroprotection of spinal neurons, sciatic nerve regeneration, and sensory-motor functional neurobehavioral parameters of nerve recovery using sciatic nerve crush injury model. Through our results, we hope to raise scientific interest into the possibility of employing Fraction B or components therefrom as therapeutic agents or as a part of a repair strategy to cure nerve injury.

## Material and Methods

### Animals

Male Wistar rats (weight 250–300 g) were obtained from the Animal Resource Center of Kuwait University Health Sciences Centre. The animals were kept under constant temperature conditions (23°C ± 2°C) and humidity, with a 12-h light/dark cycle. The rats were housed in pairs and were provided with food and water ad libitum (Renno et al., 2013, Renno et al., 2016, Renno et al., 2017; Bauder and Ferguson, 2012). Approval was obtained for all procedures from the Animal Ethics Committee of the Kuwait University Health Sciences Center, and the study was carried out per the US guidelines (NIH Publication #85-23, revised in 1985). All efforts were made to minimize the number of animals used in the study and their suffering (Renno et al., 2013, Renno et al., 2016, Renno et al., 2017; Bauder and Ferguson, 2012).

### Catfish (*Arius bilineatus*, Val.) Epidermal Protein Preparation

#### Collection of Samples at Sea

Catfish were caught on baited hook on-line in the Kuwaiti territorial waters as previously described (Al-Hassan et al., 1982). The caught fish was washed thoroughly with seawater to remove contaminants on the skin, leaving the skin-covering proteinaceous epidermal gel clean. The gel secretions (PCEGS) were collected by gently scraping the fish with a blade. The collected material was immediately frozen in dry ice and stored at −80°C until use. The PCEGS material was fractionated at 4°C to yield SPF-FB (Al-Hassan et al., 1982; Al-Hassan et al., 2020).

#### Fractionation of SPF-FB From PCEGS From the Skin of the Catfish

Fractionation of PCEGS to obtain soluble protein fraction, then Fraction B (SPF-FB) (United State Patent No.: US 10568915 B1 dated February 25, 2020) was carried out in Al-Hassan’s laboratory and was provided for this project as a frozen mixture of proteins and lipids at −80°C (Al-Hassan et al., 1991; Pace-Asciak et al., 2017; Khan et al., 2019; Al-Hassan et al., 2020).

#### Protein Assay in SPF-FB

The protein content in SPF-FB was assayed with the Coomassie Blue method (Al-Hassan et al., 1986b), and the protein concentration was calculated from the standard curve at *λ* 595 nm generated with bovine albumin, and the linear curve obtained was used to calculate total proteins in the SPF-FB.

### Experimental Groups

A total of 100 rats were used in this study. Equal numbers of animals (*n* = 20/group) were randomly assigned to the following five treatment groups: I: NAÏVE (no surgery or sciatic nerve injury); II: SHAM (SHAM-injury surgical control group); III: CRUSH (saline-treated, crushed sciatic nerve); or CRUSH + SPF-FB treated groups distributed as follows: IV: CRUSH + 0.5X SPF (IP) and V: CRUSH + 1X SPF (SC) groups. The SPF-treated animals were administered intraperitoneal (IP) (0.5X = 3 mg/kg) or subcutaneous (SC) (SPF X = 6 mg/kg) injections of once a day for 14 days starting 1 h after sciatic nerve injury (Renno et al. 2013). The SPF-FB dose was determined based on another study in our laboratory (data not shown). In a separate experiment, normal control rats (*n* = 10) were injected with 3 mg/kg of SPF-FB (IP) once daily for 12 weeks to check for SPF-FB toxicity. None of the animals died, and their blood and organs showed no side effects or toxicity (data not shown). The animals appeared healthy and grew normally in size and weight (average increased from 180 to 330 g) comparable to normal untreated matching groups. No deformity or any gross pathological alterations in all the organs were detected at the end of the experiment.

### Sciatic Nerve Crush Injury

In this study, the sciatic nerve crush injury was performed as previously described (Bauder and Ferguson, 2012; Renno et al., 2013; Renno et al., 2016; Renno et al., 2017). All approved parameters were followed to minimize animal-animal variation due to injury and to induce a standard direct trauma as previously described (Hussain et al., 2018; Renno et al., 2013; Renno et al., 2016; Renno et al., 2017; Bauder and Ferguson, 2012; Varejão et al., 2004; Varejão et al., 2004; Unal et al., 2005; Monville et al., 2006; Savastano et al., 2014). The crush-lesioned rats were randomly distributed amongst the experimental groups to minimize group variations as much as possible. Sham surgery was done for the rats in the SHAM group, where the right sciatic nerve was exposed as described above, and skin was sutured without crushing the nerve (Renno et al., 2016; Renno et al., 2017).

### Assessment of Motor and Sensory Functional Recovery

To assess motor and sensory recovery in the treated crush sciatic nerve animals, the rats in all experimental groups were evaluated for motor neurobehavioral functions 2 weeks preoperative and during 1st, 3rd, and 5th post-surgery weeks; whereas for sensory functions, the animals were tested one week preoperative and during 2nd, 4th, and 6th post-surgery weeks as described earlier (Renno et al., 2013; Renno et al., 2015; Renno et al., 2017; Hussain et al., 2018). All tests were repeated three times (with a 3–20 min interval) for each rat. The mean of three measurements was used as data for that rat for calculating the group mean for further statistical analysis. Investigators were blinded to all treatments in all experiments.

Several tests of reflexive sciatic nerve function [foot position, toe spread, extensor postural thrust (EPT), hopping, and Rotarod tests] were conducted as described in our previous studies (Bauder and Ferguson, 2012; Renno et al., 2017; Hussain et al., 2018). Briefly, EPT was measured by calculating the functional deficit; thus, the higher value indicated a poor outcome (Bauder and Ferguson, 2012; Renno et al., 2017; Hussain et al., 2018). For the hopping test, the rat was scored based on whether it hopped on the foot that was contacting the table surface (1 for hopping, 0 for no hopping) (Unal et al., 2005).

Rotarod: The rotarod test is widely used to evaluate rodents’ motor coordination, in which the animal is placed on a horizontal rod that rotates around its long axis. Rotarod performance was measured using the Rotarod test (47750 - Rat Rotarod NG, Ugo Basile SRL, Varese – ITALY). The rotarod was set at the acceleration mode (initial speed starts from 5 rpm/min; maximum speed is set at 45 rpm/min). Ten seconds after putting the rat on the rod, the acceleration was started, and the speed at which the mouse falls off was noted. The mean speed at fall was the datum used; rather than using the maximum speed, as it corrects for the extra practice, the rat received during the failed runs, which is assumed to assist performance. Time and acceleration speed were recorded (Monville et al., 2006). The Rotarod time latency at which the animal falls is indicated on the *Y*-axis.

Tactile allodynia, mechanical hyperalgesia, hotplate analgesia, and tail-flick neurobehavioral tests were conducted on all rats to assess the sensory function described as follows:

1. Tactile allodynia: The Electronic Von Frey using Ugo Basile Dynamic Plantar Aesthesiometer (37450, UGO Basile SRL, Italy) was used to evaluate the sensitivity of the skin to tactile stimulation as described previously. Mechanical allodynia thresholds from the right and left hind limbs were measured in different animal groups, and then the withdrawal reflex latencies (mean ± SD) were calculated (Bauder and Ferguson, 2012; Renno et al., 2013; Renno et al., 2017).
2. Mechanical Hyperalgesia: Paw pressure thresholds expressed in grams were measured using Analgesia Meter (Model 21025, Ugo Basile SRL, Italy). Three consecutive mechanical nociceptive threshold values were measured for each hind limb, and the mean and standard deviation (SD) of the paw pressure latencies were calculated (Renno et al., 2012; Renno et al., 2013; Renno et al., 2017; Hussain et al., 2018).
3. Thermal nociception: Thermal nociception was measured by a modified hot plate test (51°C; 35100 - Hot Cold Plate, Ugo Basile SRL, Italy). The withdrawal response threshold (WRT) defined as the time elapsed from the onset of the hotplate contact to the withdrawal of the hind paw and was characterized as a brief paw flick recorded to the nearest 0.1 s; a standard cutoff latency of 35 s was employed to prevent tissue damage (Kubo et al., 2009; Renno et al., 2017). Withdrawal within 12 s is considered a normal response. The WRT was measured for both left and right hind limbs. Each hind limb was tested thrice, with 20 min between tests to avoid sensitization. The withdrawal response latency (WRL) was calculated, and the three latencies were averaged to obtain a final result for each animal.
4. Tail flick: The spinally mediated nociceptive thresholds were determined using an Analgesia Meter Apparatus (Model 7360, UGO Basile SRL, Italy). The amount of time taken by the rat to move (flick) its tail away from the heat was recorded. A cut-off time of 30 s was used to prevent tissue damage (Kubo et al., 2009; Renno et al., 2012; Renno et al., 2013; Hussain et al., 2018).

### Statistical Analysis

All behavioral data were analyzed by 2-way repeated-measures ANOVA followed by Bonferroni’s F- and Fisher least significant difference (LSD) comparison post hoc test or Student t-test, as appropriate. All statistical analysis was performed using SPSS (version 21.0, IBM Corp.), and *p* values less than 0.05 were considered statistically significant.

### Processing of Sciatic Nerve Tissue for Light and Electron Microscopy and Quantification of Myelinated Fibers

At the end of Week 4 and Week 6 postoperative period, three animals from each experimental group were randomly selected for histopathological and morphometric evaluation, as previously described (Canan et al., 2008a; Canan et al., 2008b; Renno et al., 2013; Renno et al., 2016; Renno et al., 2017; Hussain et al., 2018).

According to principles described previously, stereological analysis of the sciatic nerves was performed by an experimenter blinded to the groups (Unal et al., 2005; Renno et al., 2017; Hussain et al., 2018). Stained sections of the sciatic nerve were digitally photographed using Olympus DP71 digital camera (Nikon COOLPIX 4500) with ×100 oil immersion objective (total magnification ×1,000). From each nerve 10 sections were selected for photography, and from each section 10 photographs were taken from randomly chosen fields. The mean total number of nerve fibers/fields in each photomicrograph was calculated as for each experimental group at Weeks 4 and 6 following sciatic nerve injury using a 1-mm^2^ counting frame previously described (Renno et al., 2017) (Supplementary Figure S1). Quantification was done by Image-Pro Plus image analyzing software (Version 6.0.0.260 for Windows 2000/XP Professional; Media Cybernetics) (Francisco et al., 2004). The unbiased counting frame method with a square sample area (900 μm^2^) was used to obtain an estimation of the total axon number impartially from nerve cross-section (Francisco et al., 2004; Renno et al., 2017). A counting frame was placed on the image, and the sampling area was selected in a systematic, uniform random manner (Kaplan et al., 2005). A mean sampling of each sectioned nerve profile was done in 70 × 70 µm step size systematically. This ensured that all locations within a nerve cross-section (1 μm-thickness) were equally represented and that all axon profiles were sampled with equal probability regardless of shape, size, orientation, and location (Varejão et al., 2004; Varejão et al., 2004; Turgut et al., 2010) (Supplementary Figure S1). The mean cross-sectional areas and mean axonal perimeters of myelinated axon were obtained from 10 different and randomly selected samples from each animal (5 animals/group). The averaged cross-sectional areas and averaged perimeters of myelinated axons measured in square micrometers (µm^2^) were calculated for each experimental group at Weeks 4 and 6 following sciatic nerve injuries. Further confirmation of the morphometric findings was attained from the axon and nerve fiber diameter distribution at Week 4 and Week 6. The percentage frequency of the myelinated axons and nerve fibers (*y*-axis) were plotted against the diameter sizes (*x*-axis).

### Morphometric and Stereological Analyses Leading to Estimation of Myelin Sheath Thickness

Images were viewed on a 15-inch Samsung SyncMaster 500S color monitor. The axon (d) and the nerve fiber diameters (D) were measured automatically using Image-Pro Plus 6.0 image analysis software and were used to calculate myelin thickness. The thickness measurements acquired from all sampled axons were then averaged to obtain the mean myelin thickness (m = [D − d]/2), as was previously detailed (Renno et al., 2017; Hussain et al., 2018). The axon’s g-ratio is a measure of myelin thickness calculated by dividing the axonal diameter by the total diameter of the axon plus myelin sheath (nerve fiber diameter). This indicates the level of remyelination in axons that are treated with SPF Fraction B.

### Data Analysis

Images of the distal portions of the right sciatic nerve obtained from three rats per group were analyzed. A total of 10 images from each section were digitized and stored in uncompressed tagged image file format (TIFF) with 24-bit RGB class and 640 × 480-pixel resolution. The 10 sampling results per animal were averaged then the averages of five animal data per group were statistically analyzed. All data are presented as the mean ± standard deviation of the mean (SEM). All calculations and all statistical procedures were performed using SPSS (version 21.0). The group values were compared using a Mann-Whitney *U*-test. A *p*-value of less than 0.05 was considered significant.

### Protein Quantification of Myelin Basic Protein (MBP), Glial Fibrillary Acidic Protein (GFAP) and Growth Associated Protein 43 (GAP-43)

Western blot was performed thrice for each rat, as described previously (Renno et al., 2016; Renno et al., 2017; Hussain et al., 2018). All bands labeled with the anti-MBP antibody, GFAP antibody, and GAP-43 antibody (*n* = 5 sciatic nerves/group) were scanned, and their density was quantified by Densitometer GS-800, normalized with the actin band density.

### Processing of Spinal Cord Tissue for NeuN, GFAP and GAP-43 Immunostaining and Quantification

While collecting the sciatic nerve, lumbar spinal cords were dissected and fixed by immersion overnight at 4°C, in a mixture of 4% paraformaldehyde and 0.1% glutaraldehyde in 0.1 M phosphate buffer, pH 7.4 (Renno et al., 2017; Hussain et al., 2018). Every 15th section of the spinal cord was stained for Cresyl Violet staining and immunostained for neurons using Primary antibodies for NeuN, GFAP and GAP 43. 5 µm thick paraffin sections of the spinal cord, were cut and mounted on Poly-L-lysine coated slides and kept overnight for drying. The tissue sections were deparaffinized in xylene and rehydrated in a graded series of alcohol and then taken to the water. Endogenous peroxidase activity was quenched by treating sections with 3% hydrogen peroxide for 15 min, followed by 30 min incubation in 50 mM glycine and 0.1% sodium borohydride and then washed with PBS (Ph 7.4). Non-specific binding of antibodies was blocked by treating the sections with 5% normal goat serum. Sections were incubated with Primary antibodies (Anti-NeuN, Clone A60, Mouse Monoclonal Antibody, Cat# MAB377, Millipore. Billerica, Massachusetts, United States), Anti-GFAP antibody (GA-5: sc-58766, Mouse Monoclonal – Santa Cruz Biotechnology) or Anti-GAP-43 Antibody (B-5: sc-17790 – Santa Cruz Biotechnology) overnight at 4°C. Sections were then washed with PBS and treated with biotinylated goat anti-mouse IgG [(1:200), Vector Labs, Burlingame, CA, United States)] and 1% normal goat serum for 2 h at room temperature. Slides were then washed three times with PBS and treated with Avidin-Biotin Complex (Vector Labs, PK-6200, Burlingame, CA, United States) along with 0.1% Tween 20 for 1 h at room temperature. Sections were color developed with 3-diaminobenzidine as a chromogen (DAB kit, SK-4100, Vector Labs, Burlingame, CA, United States), for 30 s or until the desired brown color was obtained as seen under the microscope. Then slides were washed with distilled water, counterstained in hematoxylin for 5 min, followed by bluing under tap water for 5 min. The slides were dehydrated in graded ethanol and cleared in xylene. Finally, a coverslip was mounted on top of sections using DPX mountant for histology. For Cresyl violet staining, sections were mounted on gelatin-coated slides, air-dried overnight. Sections were hydrated in graded ethanol and stained with 0.1% cresyl violet stain. Slides were dehydrated in graded ethanol and cleared in xylene and mounted with DPX mountant for histology (44581, Sigma) (Renno et al., 2017; Hussain et al., 2018).

The number of NeuN labeled neurons in the ventral and dorsal grey horns of the spinal cord were counted using Cell Sens Dimension software. From each rat, ten sections were selected for neuron quantification. The spinal cord region under analysis was focused at ×40 magnification, and an image was transferred to a computer monitor with a high-resolution digital Nikon camera attached to an Olympus microscope (DP-72). The total number of neurons in the entire ventral and dorsal grey horns were counted. Slides were coded to avoid observers’ bias. The mean number of neurons per section was calculated for statistical analysis (Renno et al., 2015; Renno et al., 2016; Hussain et al., 2018).

The GFAP and GAP-43 immunoreactive staining intensity was measured with Cell Sens Dimension software. From each rat, ten sections were randomly selected for intensity measurement. The 10 sampling results per animal were averaged then the averages of five animal data per group were statistically analyzed. The total intensity in the entire ventral and dorsal grey horns was measured, and the mean GFAP and GAP-43 immunoreactive intensity per section was calculated for statistical analysis (Renno et al., 2015; Renno et al., 2016; Hussain et al., 2018).

### Statistical Analysis

The NeuN, GFAP and GAP-43 immunohistochemical data were analyzed by one-way ANOVA followed by Bonferroni’s F-test and Fisher’s least significant difference (LSD) comparison post hoc test to determine differences in individual baseline values. All statistical analysis was performed using SPSS (version 21.0, IBM Corp.); *p* values less than 0.05 were considered statistically significant (Renno et al., 2016; Renno et al., 2017; Hussain et al., 2018).

## Results

SHAM animals’ hind paws displayed a healthy appearance following the surgical procedure. Also, the animals’ gait was not hampered by the skin and muscle wounds. The CRUSH group’s hind paw foot and toes were strongly flexed during the first week. The rats were also incompetent at standing on their operated hind paw. The crush group displayed partial weight-bearing starting from the second week. In contrast, the SPF-FB-treated rats exhibited noticeable enhancement in the clinical representation and weight-bearing over the following weeks compared to the CRUSH animals.

### Behavioral Tests

**FIGURE 1:**
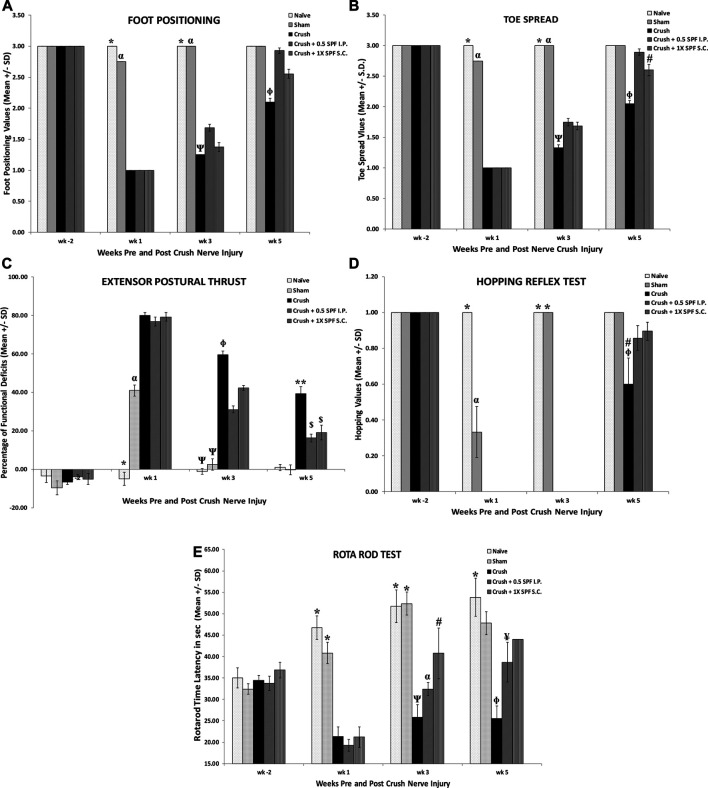
**A)**: Sciatic nerve crush produced a significant decrease in foot position values in the CRUSH + SALINE group at week 1, week 3 and week 5 post-surgery weeks compared to NAÏVE and SHAM groups. IP and SC SPF treatments significantly increased the foot position values throughout the experiment compared to the CRUSH + SALINE group. * indicates *p* < 0.0001 NAÏVE vs. CRUSH and treated groups; α indicates *p* < 0.0001 SHAM vs. CRUSH and treated groups; Ψ indicates CRUSH *p* < 0.001 vs. CRUSH + 0.5X SPF-FB (IP); Φ indicates CRUSH *p* < 0.001 vs. treated groups. **(B)**: Analysis of toe spread outcome. The CRUSH + SPF-FB rats showed both clinically and statistically significant recovery in toe spread following sciatic nerve injury. Similar to the foot position, recovery of the toe spread started at the end of week 3 and continued until the end of week 5. * indicates *p* < 0.001 NAÏVE vs. CRUSH and treated groups; α indicates *p* < 0.0001 SHAM vs. CRUSH and treated groups; Ψ indicates *p* < 0.001 CRUSH vs. all groups; Φ indicates *p* < 0.0001 CRUSH vs. ALL groups; # indicates *p* < 0.04 CRUSH + 0.5 SPF-FB (IP) vs. NAIVE and SHAM. (**C)**: The CRUSH + SPF-FB-treated groups displayed an early decease in EPT at week 3 post-nerve injury. * indicates *p* < 0.0001 NAÏVE vs. All groups; α indicates *p* < 0.0001 SHAM vs. All groups; Ψ indicates *p* < 0.0001 NAÏVE and SHAM groups vs. CRUSH and All SPF-FB-treated groups; Φ indicates *p* < 0.0001 CRUSH vs. 0.5X SPF-FB (IP) and 1X SPF-FB (S.C.)-treated groups; ** indicates *p* < 0.0001 CRUSH vs. All groups; $ indicates *p* < 0.0001 the treated groups vs. all other groups. (**D):** The CRUSH + SPF-FB-treated groups displayed an early hopping response at week 5 post-nerve injury. Meanwhile, CRUSH animals showed a significantly less percentage of recovered hopping reflex. * indicates *p* < 0.0001 NAÏVE and SHAM vs. CRUSH and CRUSH + SPF-FB-treated groups; α indicates *p* < 0.0001 SHAM vs NAÏVE, CRUSH and SPF-FB-treated groups; # indicates *p* < 0.03 CRUSH vs. CRUSH + 0.5 SPF-FB (IP); Φ indicates *p* < 0.004 CRUSH vs. NAÏVE and SHAM and sham; # indicates *p* < 0.0001 CRUSH vs. the treated groups; Φ indicates *p* < 0.0001 CRUSH vs. NAÏVE and SHAM. (**E)**: The CRUSH + 0.05X SPF-FB (IP)-treated groups displayed an increase in Rota rod latency at week 3 post-nerve injury. * indicates *p* < 0.0001 NAÏVE and SHAM vs. CRUSH and CRUSH + SPF-FB-treated groups; α indicates *p* < 0.0001 vs. CRUSH and SPF-FB-treated groups; Ψ indicates *p* < 0.004 CRUSH vs. CRUSH + 1X SPF-FB (S.C.); # indicates *p* < 0.01 CRUSH + 1X SPF-FB (S.C.) vs. SHAM; Φ indicates *p* < 0.004 CRUSH vs. NAÏVE and SHAM and CRUSH + 1X SPF-FB (S.C.); ¥ indicates *p* < 0.001 CRUSH + 0.5X SPF-FB (IP) vs. CRUSH. All values are expressed as means ± SDs (error bars).

Sciatic nerve crush produced a significant (*p* < 0.0001) decrease of foot position values in the CRUSH + SALINE group at week 1, week 3, and week 5 post-surgery weeks compared to NAÏVE and SHAM groups (Figure 1A). 0.5X IP and 1X SC. SPF-FB-treatments significantly (*p* < 0.001) increased the foot position values at Week 3 compared to the CRUSH + SALINE group. Likewise, the mean values of the foot position analysis were significantly (*p* < 0.0001) higher in CRUSH + 0.5X IP SPF-FB-treated at Week 5 (Figure 1A). The CRUSH + 0.5X IP SPF-FB-treated animals’ mean values of the foot position were similar to NAÏVE and SHAM groups. Like the foot position, significant recovery (*p* < 0.0001) of the toe spread started at the end of week 3 in the CRUSH + 0.5X IP and CRUSH + 1X SC SPF-FB-treated groups (Figure 1B). At the end of Week 5, the CRUSH + 0.5X IP SPF-FB-treated group demonstrated significant (*p* < 0.0001) foot position recovery compared to CRUSH animals. However, the improvement of CRUSH + 0.5X IP SPF-FB-treated animals was still lagging (*p* < 0.04) behind the NAÏVE and SHAM groups (Figure 1B).

Figure 1C shows the functional recovery evaluations following crush injury and IP and SC SPF-FB treatments. The SHAM and the SPF-FB-treated groups displayed significant (*p* < 0.0001) EPT deficits (approximately 40% deficits) at Week 1 following crush injury. Further, the CRUSH and SPF-FB-treated groups lost significant (*p* < 0.0001) EPT (approximately 80% deficits) compared to NAÏVE animals at Week 1 following crush injury. At Week 3 post-injury, the CRUSH + SPF-FB-treated groups displayed a significant (*p* < 0.0001) decrease in EPT compared to control groups. However, the SPF-FB-treated groups displayed more than 85% improvement in motor EPT recovery (*p* < 0.0001) compared to CRUSH (approximately 60% recovery) by the end of Week 5 following nerve injury.

The results of the hopping test (Figure 1D) demonstrated significant (*p* < 0.0001) early recovery in the SHAM animals compared to the NAÏVE group at the end of Week 1. However, the CRUSH + SPF-FB-treated animals did not show any hopping response at Week 1 and Week 3. At Week 5 post-nerve injury, the CRUSH and CRUSH + 0.5X SPF-FB(IP)-treated group demonstrated hopping response (approximately 60 and 90%, respectively). The hopping response of the SPF-FB-treated group was similar to the NAÏVE and SHAM groups and statistically (*p* < 0.03) much higher than CRUSH animals at Week 5 (Figure 1D).

The Rotarod acceleration mode performance test (Figure 1E) displayed a significant decrease in the time latency of the CRUSH and CRUSH + SPF-FB-treated groups (approximately 20 s) compared to NAÏVE and SHAM animals (nearly 50 and 40 s, respectively) at Week 1 post-nerve injury. At Week 3 following the surgery, the CRUSH + 1X SPF-FB (SC) (*p* < 0.004) demonstrated higher time latency on the Rotarod compared to the CRUSH group. Meanwhile, the Rotarod performance of the SPF-FB-treated animals displayed significantly (*p* < 0.001) higher time latency (approximately 40–45 s) compared to the CRUSH group (nearly 25 s) at Week 5. The SPF-FB-treated groups showed no significant difference in time latency to the SHAM group (approximately 47 s).

**FIGURE 2:**
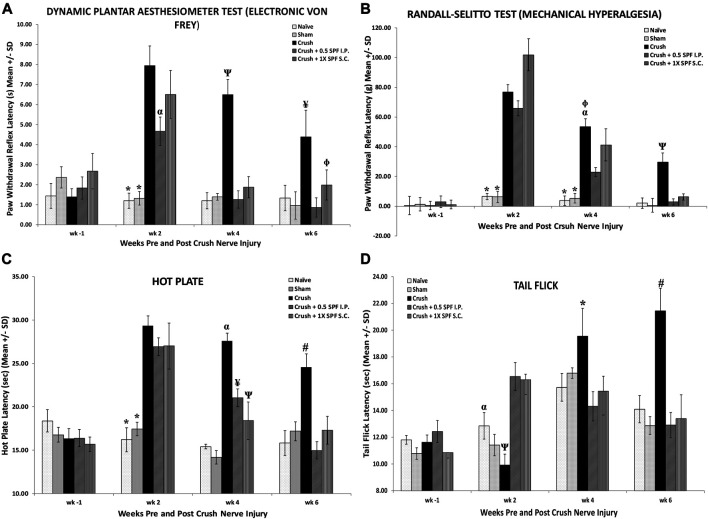
(**A)**: Bar graphs showing tactile allodynia. The CRUSH and the CRUSH + SP-FB-treated groups showed a significant decrease in analgesia planter test at week 1 post-injury. By week 3, the CRUSH, CRUSH + 1X SPF-FB (S.C.) and CRUSH + 1X SPF-FB (IP) groups displayed an increase in analgesia. However, CRUSH + 0.5X SPF-FB (IP) group showed a significant decrease compared to CRUSH and CRUSH + SPF-FB S.C.-treated animals. The CRUSH + 0.5X SPF-FB (IP) stayed significantly different from the CRUSH group at week 5 following nerve injury. * indicates *p* < 0.0001 NAÏVE and SHAM vs. CRUSH, CRUSH + 0.5X SPF-FB (IP) and CRUSH + 1X SPF-FB (S.C.); ^α^ indicates *p* < 0.02 CRUSH + 0.5X SPF-FB (IP) vs. CRUSH; Ψ indicates *p* < 0.0001 CRUSH vs. NAÏVE, SHAM, and treated groups; Φ indicates *p* < 0.05 CRUSH + 1X SPF-FB (S.C.) vs. CRUSH; ¥ indicates *p* < 0.02 CRUSH vs. NAÏVE, SHAM and CRUSH + 0.5X SPF-FB (IP). (**B)**: The CRUSH + SPF-FB-treated groups showed a significant decrease in paw withdrawal reflex latency periods compared with the CRUSH group at week 4. At week 6, CRUSH + 0.5X SPF-FB(IP)-treated animals displayed a significant decrease in paw pressure latency. * indicates *p* < 0.0001 NAÏVE and SHAM vs. Crush and treated groups; α indicates *p* < 0.0001 CRUSH vs. CRUSH + 0.5X SPF-FB (IP); Φ indicates *p* < 0.01 CRUSH vs. CRUSH + 1X SPF-FB (S.C.); Ψ indicates *p* < 0.0001 CRUSH vs. NAÏVE, SHAM and CRUSH + 0.5X SPF-FB (IP). **(C)**: Nerve crush injury produced a severe nociception deficit in the CRUSH and CRUSH-SPF-FB-treated groups at week two following nerve injury. The CRUSH- SPF-treated groups showed a significant thermal nociceptive recovery starting at Week 4. At Week 6 post-injury, the CRUSH + 0.5X SPF-FB (IP) animals maintained the thermal nociception recovery comparable to control groups. * indicates *p* < 0.0001 NAÏVE and SHAM vs. CRUSH and CRUSH + SPF-FB-treated groups; α indicates *p* < 0.0001 CRUSH vs. NAÏVE, SHAM and CRUSH + SPF-treated groups; ¥ indicates *p* < 0.005 CRUSH + 0.5X SPF-FB(IP) vs. SHAM and CRUSH; Ψ indicates *p* < 0.01 CRUSH + 1X SPF-FB(S.C.) vs. NAÏVE and SHAM groups; # indicates *p* < 0.001CRUSH vs. NAÏVE, SHAM and treated groups. **(D)**: The CRUSH + 0.5X SPF-FB-treated group showed significant tail-flick withdrawal latency recovery compared CRUSH group by the end of week 4 post-surgery. This nociception normalization was sustained until the end of the experiment, whereas CRUSH animals continued to display a significant increase in the tail-flick withdrawal latency compared to controls and SPF-FB-treated groups. * indicates *p* < 0.01 CRUSH vs. all groups; α indicates *p* < 0.01 SHAM vs. CRUSH + 0.5X SPF-FB(IP) and CRUSH + 1X SPF-FB(S.C.); Ψ indicates *p* < 0.001 CRUSH vs. CRUSH + SPF-FB-treated groups; # indicates *p* < 0.0001 CRUSH vs. all groups. All values are expressed as means ± SDs (error bars).

We performed several neurobehavioral sensory tests to evaluate the effect of SPF-FB on the sensory activities following nerve injury. The CRUSH and the CRUSH + SPF-FB- treated groups showed a significant (*p* < 0.0001) increase in allodynia test at Week 2 post-injury (Figure 2A). However, the SPF-FB-treated groups showed a significant (IP *p* < 0.0001) and SC *p* < 0.008) decrease in allodynia reflex latency compared to the CRUSH group by Week 4 post-injury. By week 6, the lessening in allodynia reflex latency stayed significantly (*p* < 0.02) low in SPF-FB-treated rats compared to the CRUSH group.

Likewise, using the paw pressure latency test (Figure 2B), the CRUSH and the CRUSH + SPF-FB treated groups showed a significant (*p* < 0.0001) increase in mechanical hyperalgesia test at Week 2 and Week 4 post-injury compared to NAÏVE and SHAM groups. However, the CRUSH + 0.5X SPF-FB (IP) and CRUSH + 1X SPF-FB (SC) treated groups showed a significant (*p* < 0.0001, *p* < 0.01, and *p* < 0.01, respectively) decrease in the paw withdrawal reflex latencies compared to the CRUSH group at Week 4. At Week 6 following nerve injury, SPF-FB-treated animals displayed a significant (*p* < 0.0001) decrease in the paw pressure latency indicating back to normal mechanical hyperalgesia values (Figure 2B).

Nerve crush injury produced a significant (*p* < 0.0001) delay in the hot palate latency in the CRUSH and CRUSH + SPF-FB-treated groups at Week 2 (Figure 2C). The CRUSH + SPF-FB-treated groups showed a significant (*p* < 0.0001) thermal nociceptive recovery starting in Week 4. At week 6 post-injury, the SPF-FB-treated groups maintained the thermal nociception recovery comparable to control groups. The CRUSH group displayed significant (*p* < 0.001) higher hot plate latency compared to NAÏVE, SHAM, and CRUSH + SPF-FB-treated groups at Week 6 following nerve injury (Figure 2C).

Similarly, the time course of tail-flick withdrawal latencies (Figure 2D) shows the fluctuation in tail-flick withdrawal latencies among the various groups at Week 2 and Week 4 following surgery. The CRUSH + SPF-FB-treated groups showed significantly (*p* < 0.01) tail-flick withdrawal latency recovery compared to the CRUSH group by the end of Week 4 post-surgery. This central nociception normalization was sustained until the end of the experiment. In contrast, CRUSH animals continued to display a significant (*p* < 0.0001) increase in the tail-flick withdrawal latency compared to controls and CRUSH + 0.5X SPF-FB (IP) groups. Notably, there was no difference between the SPF-FB-treated groups and the NAÏVE and SHAM groups at Week 6 post nerve injury (Figure 2D). In brief, SPF-FB treatment of rats with sciatic nerve crush injury produced significant improvement in the motor and sensory behavioral tests compared to control groups.

### Histological and Morphometric Analysis

#### Light Microscopy

**FIGURE 3:**
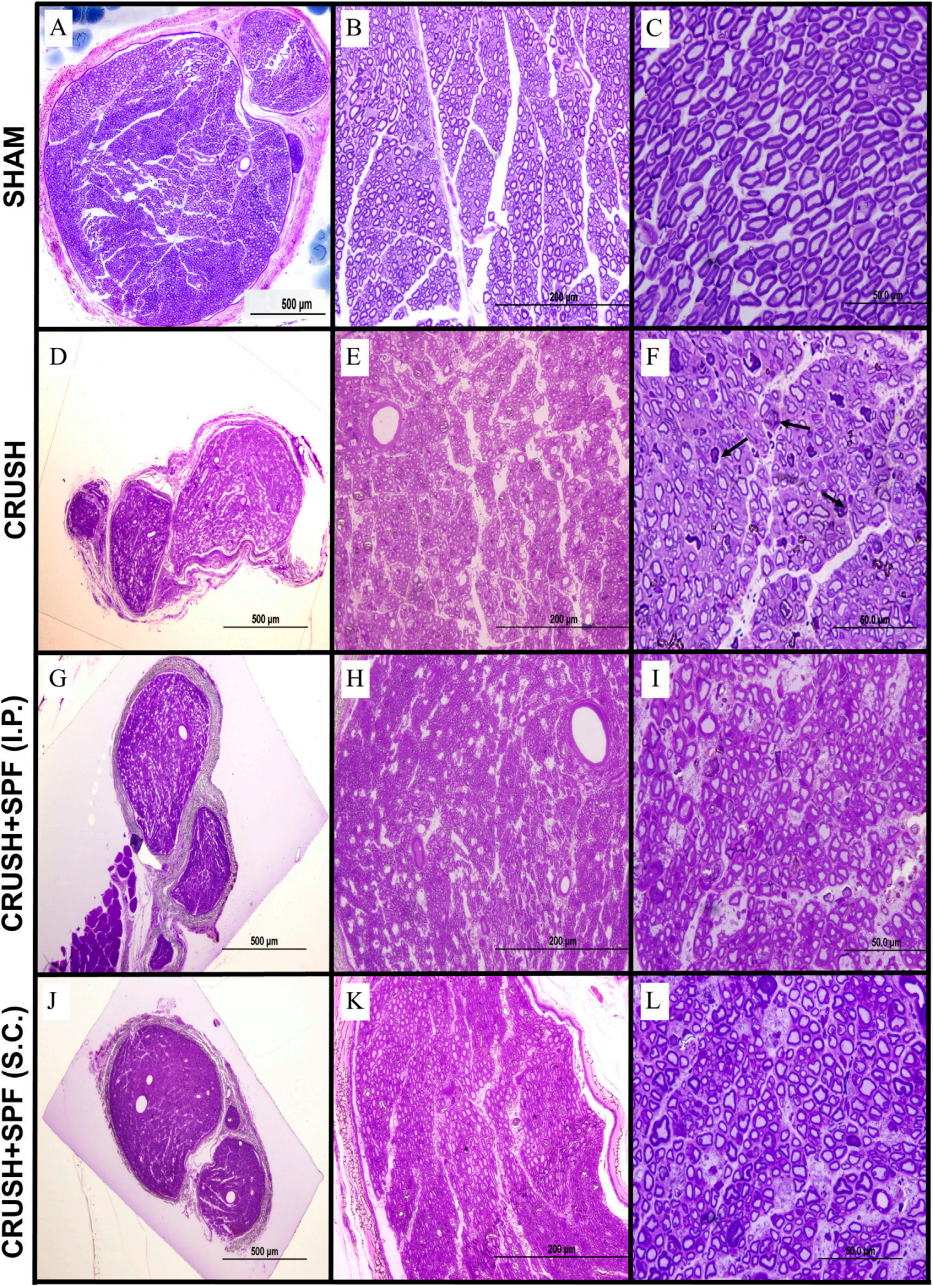
Toluidine blue-stained photomicrographs of semi-thin transverse sections of sciatic nerves obtained from animals in the SHAM (**A–C**), CRUSH (**D–F**), CRUSH + 0.5X SPF-FB(IP) (**G–I**) and CRUSH + 1X SPF-FB(S.C.) (**J–L**) groups at week 6 following nerve injury (Column I; ×100, Column II, ×400 and Column III, ×1,000). Although sciatic nerves from CRUSH (**D–F**) animals display regenerative recovery but still show the presence of smaller mini-fascicles nerve fibers with thin myelin sheaths and more myelin configurations and debris (arrows) at week 6 following crush injury, in contrast, sciatic nerve sections of and CRUSH + 0.5X SPF-FB(IP) (**G–I**) and CRUSH + 1X SPF-FB (S.C.) (**J–L**) groups show remarkable nerve regeneration with less myelin debris compared to those from the CRUSH (**D–F**) group. Note that the SPF-FB-treated nerves show more compactly arranged, regularly shaped, and more myelin surrounding the axons compared to the CRUSH group.

Histological examination of sciatic nerve sections from the SHAM group at Week 4 (Supplementary Figure S2) and Week 6 (Figures 3A–C) revealed a regular nerve appearance at different magnifications. Wallerian degeneration and unmyelinated fibers were evident in the tissue sections of the sciatic nerve 10 mm distal to CRUSH groups’ lesion site at Week 4 (Supplementary Figure S2). The CRUSH nerves showed smaller mini-fascicles nerve fibers with less myelin and macrophages filled with degraded myelin following crush injury. At week 6 following crush injury, nerves from CRUSH animals (Figures 3D–F) displayed regenerative recovery but still showing smaller mini-fascicles nerve fibers, thin myelin sheaths, and more myelin configurations and debris.

In contrast, at Week 4 following injury, sciatic nerve sections of CRUSH + 0.5X SPF-FB (IP) and CRUSH + 1X SPF-FB (SC) groups showed remarkable nerve regeneration with large-size nerve fibers surrounded with noticeable increase in myelin layers compared with those from the CRUSH (Supplementary Figure S2). The myelin debris and macrophages in the SPF-FB-treated groups were much less compared to CRUSH nerves. Likewise, at Week 6, sciatic nerve sections of CRUSH + 0.5X SPF-FB (IP) (Figures 3G–I) and CRUSH + 1X SPF-FB (SC) (Figures 3J–L) groups showed remarkable nerve regeneration with less myelin debris compared to those from the CRUSH group. Further, the SPF-FB-treated nerves showed more compactly arranged, regularly shaped, and more myelin surrounding the axons than the CRUSH group.

#### Transmission Electron Microscopy

**FIGURE 4:**
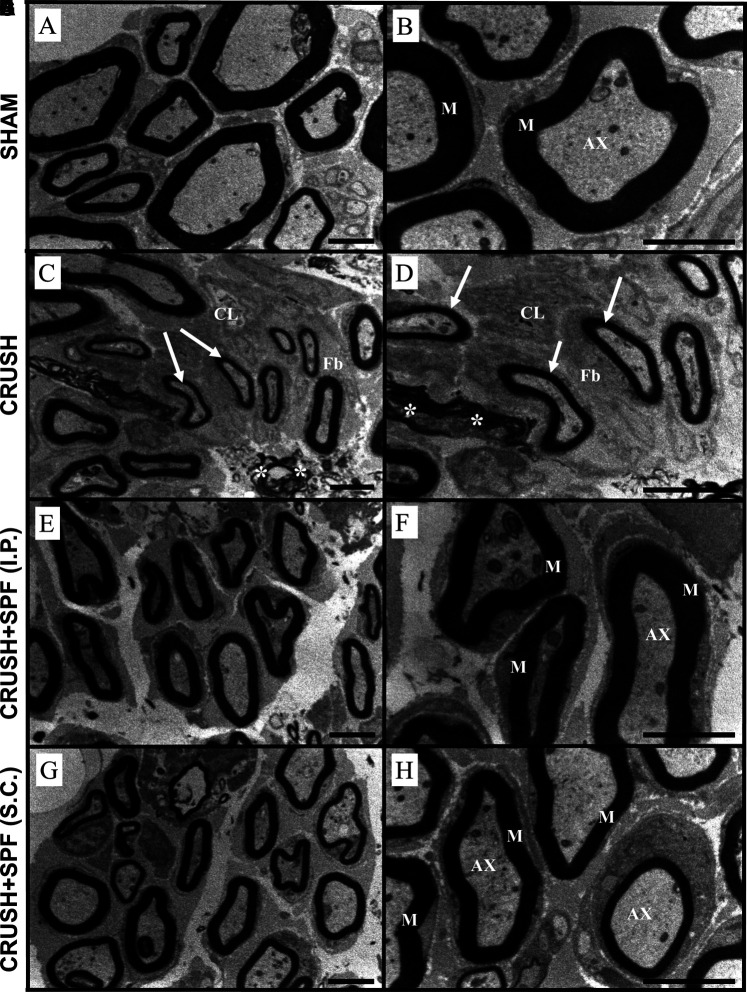
Electron micrographs of the sciatic nerve from the SHAM **(A,B)**, CRUSH **(C,D)**, and CRUSH + SPF-FB(IP) **(E,F)** and CRUSH + SPF-FB(S.C.) **(G,H)** groups at week 6 post-injury. Note that the majority of the axons and nerve fibers are still small in size and thinly myelinated (arrows) with a noticeable amount of extracellular cells (CL) and collagen fibers (Fb) fibers along with disintegrated and remnants of myelin scattered in between the axons (asterisk) in CRUSH nerves. SPF-FB -treated crushed sciatic **(E,G,H)** nerves showed normal, healthy appearing myelin sheaths, with normal thickness and normal apparent axons. M-myelin sheath; AX- axons. Scale bar = 1 µm.

Electron micrographs of the sciatic nerve of CRUSH animals showed enormously irregular fashioned and extremely condensed irregular myelin sheaths, fragmented axonal fibers, and myelin fragments scattered in between the axons at Week 4 following injury compared to SHAM samples (Supplementary Figure S3). Also, the CRUSH samples displayed a sizeable number of unmyelinated or slightly myelinated axons. At Week 6 (Figures 4C,D), the majority of the axons and nerve fibers were still small in size and thinly myelinated with a noticeable amount of extracellular cells and collagen fibers along with disintegrated and remnants of myelin scattered in between the axons in CRUSH nerves compared to SHAM group (Figures 4A,B). SPF-FB-treated nerves showed normal Schmidt-Lantermann clefts associated with normal and healthy appearing myelin sheaths, with normal thickness and normal apparent axons at Week 4 (Supplementary Figure S3) as well as at Week 6 (Figures 4E–H). Also, the Schwann cells appeared normal in SPF-FB-treated animals. SPF-FB-treated animals displayed newly regenerated collagen fibers like normal collagen fibers with well-organized distribution and absence of disintegrated myelin figures in the extracellular matrix. To summarize, crush-injured sciatic nerves treated with 0.5X SPF-FB displayed remarkable normal histological appearance at the light and electron microscopic levels compared to control nerves.

### Stereological Analysis

**FIGURE 5:**
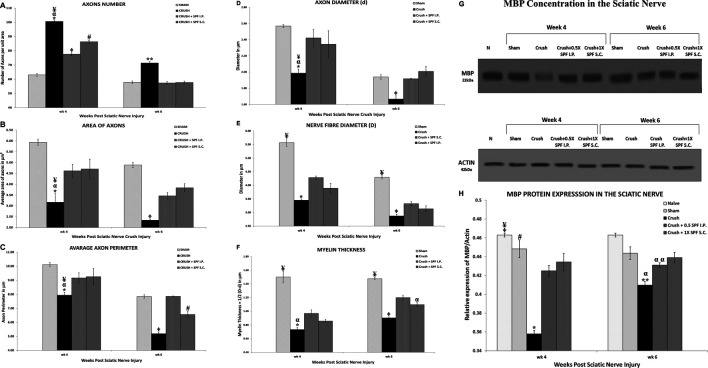
**(A)**: Number of axons/field (at ×1,000 magnification) in a cross-section of the sciatic nerve distal to the injury site. Note SPN treatments significantly decreased the number of axons/field following nerve crush injury at Week 4. Further, the number of axons/field of the SPF-FB-treated groups returned to the SHAM level by Week 6. * indicates *p* < 0.0001 CRUSH vs. SHAM; α indicates *p* < 0.001 CRUSH vs. CRUSH + 0.5X SPF-FB(IP); ¥ indicates *p* < 0.01 CRUSH vs. CRUSH + 1X SPF-FB(S.C.); Φ indicates *p* < 0.02 CRUSH + 0.5X SPF-FB (IP) vs. SHAM; # indicates *p* < 0.001 CRUSH + 1X SPF-FB(S.C.) vs. SHAM; ** indicates *p* < 0.005 CRUSH vs. all groups. **(B)**: The Crush + SPF-FB animals showed a significant increase in the mean cross-sectional areas of the myelinated axons at Week 4 and Week 6 compared with the Crush group. * indicates *p* < 0.002 CRUSH vs. SHAM; α indicates *p* < 0.01 CRUSH vs. CRUSH + 0.5X SPF-FB (IP); ¥ indicates *p* < 0.05 CRUSH vs. CRUSH + 1X SPF-FB(S.C.); Φ indicates *p* < 0.01 CRUSH vs. all groups. **(C)**: The CRUSH + SPF-FB-treated animals showed a significant increase in the mean cross-sectional areas of the myelinated axons at Week 4 and Week 6 compared to the CRUSH group. * indicates *p* < 0.002 CRUSH vs. SHAM; α indicates *p* < 0.02 CRUSH vs. CRUSH + 0.5X SPF-FB(IP); ¥ indicates *p* < 0.03 CRUSH vs. CRUSH + 1X SPF-FB(S.C.); Φ indicates *p* < 0.0001 CRUSH vs. all groups; # indicates *p* < 0.003 CRUSH + 1X SPF-FB(S.C.) vs. CRUSH + 0.5X SPF-FB(IP). **(D–F)**: Stereological estimation of the myelinated axon diameter **(D)**, myelinated nerve fiber diameter **(E),** and myelin thickness **(F)** obtained from 10 different random samples from each group (n = 5). **(D)**: The CRUSH animals displayed a significant decrease in axon diameter at all the time points assessed following nerve injury compared with SHAM and CRUSH + SPF-FB-treated groups. The CRUSH + SPF-FB-treated group showed no significant change in comparison with SHAM animals at Week 4 and Week 6. * indicates *p* < 0.002 CRUSH vs. SHAM; α indicates *p* < 0.01 CRUSH vs. CRUSH + 0.5X SPF-FB(IP); ¥ indicates *p* < 0.03 CRUSH vs. CRUSH + 1X SPF-FB(S.C.); Φ indicates *p* < 0.03 CRUSH vs. All groups. **E:** Likewise, the CRUSH group displayed a significant decrease in nerve fiber diameter at all the time points assessed following nerve injury compared with SHAM and CRUSH + 0.5X SPF-FB(IP) groups. * indicates *p* < 0.001 CRUSH vs. CRUSH + 0.5X SPF-FB(IP); ¥ indicates *p* < 0.0001 SHAM vs. All treated groups; Φ indicates *p* < 0.04 CRUSH vs. CRUSH + 0.5X SPF-FB(IP). **(F)**: The calculated myelin thickness for the CRUSH and CRUSH + SPF-treated groups show a significant decrease in myelin thickness compared to SHAM animals at Week 4 and Week 6 following sciatic nerve injury. However, the myelin thickness in CRUSH + SPF-treated animals increased significantly at Week 4 and became more at Week 6 compared with the CRUSH group. ¥ indicates *p* < 0.01 SHAM vs. All groups; * indicates *p* < 0.003 CRUSH vs. CRUSH + 0.5X; SPF-FB(IP); α indicates *p* < 0.05 CRUSH and CRUSH + 1X SPF-FB(S.C.) vs. CRUSH + 0.5X SPF-FB(IP); Φ indicates *p* < 0.007 CRUSH vs. CRUSH + 0.5X SPF-FB(IP) and CRUSH + 1X SPF-FB(S.C.). Values are expressed as means and SDs (*error bars*). **(G)**: Western blot analysis of myelin basic protein (MBP) in the sciatic nerves from the experimental groups (n = 5) at Week 4 and Week 6 following nerve injury. Note thick MBP bands in the CRUSH + SPF-FB-treated group. **(H)**: Bar graph shows the MBP density in distal nerve samples normalized with actin band densities. Note significantly increased basic myelin content in the SPF-treated group at Week 4 and Week 6 post sciatic nerve injury. * indicates *p* < 0.0001 CRUSH vs. All groups; Φ indicates *p* < 0.02 NAÏVE vs. CRUSHv0.5X SPF-FB(IP); ¥ indicates *p* < 0.0001 NAÏVE vs. CRUSH + 1X SPF(S.C.); # indicates *p* < 0.02 SHAM vs. CRUSH + 0.5X SPF(IP); ** indicates *p* < 0.0001 CRUSH vs. NAÏVE and SHAM; α indicates *p* < 0.01 CRUSH vs. treated groups; α α indicates *p* < 0.0001 CRUSH + 0.5X SPF-FB(IP) vs. NAÏVE. All values are expressed as means + SDs (error bars).

Figure 5 illustrates the morphometric data analysis of sciatic nerves after 4- and 6-weeks post-surgery. The mean total number of the myelinated axons at Weeks 4 and 6 following nerve injury was calculated using the square counting frame (Figure 5A). At Week 4, the CRUSH + 0.5X SPF-FB (IP)-treated (*p* < 0.02) CRUSH + 1X SPF-FB (S.C.)-treated (*p* < 0.001) and CRUSH (*p* < 0.0001) groups showed a significant increase in the mean total myelinated axons numbers compared with SHAM groups. IP and SC. SPF-FB treatments significantly (*p* < 0.001 and *p* < 0.01, respectively) decreased the number of axon unit areas following nerve crush injury at Week 4 compared to the CRUSH group. Further, the number of axons per unit area of the IP and SC. SPF- FB-treated groups returned to the SHAM level by Week 6. However, in the CRUSH animals, the number of axons per unit area remained significantly (*p* < 0.005) increased compared to SHAM and IP, and SC. SPF- FB-treated groups (Figure 5A).

Figure 5B shows the mean cross-sectional areas in square micrometers (µm^2^) for each rat in Week 4 and 6 following nerve injuries. The CRUSH animals showed significant reduction in the average areas of myelinated axons compared with SHAM (*p* < 0.002), CRUSH + 0.5X SPF-FB (IP) (*p* < 0.01), CRUSH vs. CRUSH + 1X SPF-FB (SC) (*p* < 0.05) at Week 4. In contrast, IP and SC SPF- FB-treated groups revealed a significant (*p* < 0.01) increase in the myelinated axons’ mean areas compared to the CRUSH group at Week 6. Moreover, IP and SC SPF-FB-treated animals showed no difference in mean areas compared to SHAM animals (Figure 5B).

The averaged perimeters of myelinated axons measured in square micrometers (µm^2^) in the CRUSH animals showed significant decrease compared with SHAM (*p* < 0.002), CRUSH + 0.5X SPF-FB(IP) (*p* < 0.02) vs. CRUSH + 1X SPF-FB(S.C.) (*p* < 0.03) at Week 4 (Figure 5C). At Week 6, the IP and SC. SPF-FB-treated groups displayed a significant (*p* < 0.0001) increase in the myelinated axons’ mean areas compared to the CRUSH group. However, the average perimeters of myelinated axons in the CRUSH + 1X SPF-FB(SC) group was still significantly (*p* < 0.003) lower than CRUSH + 0.5X SPF-FB(IP) group.

**FIGURE 6:**
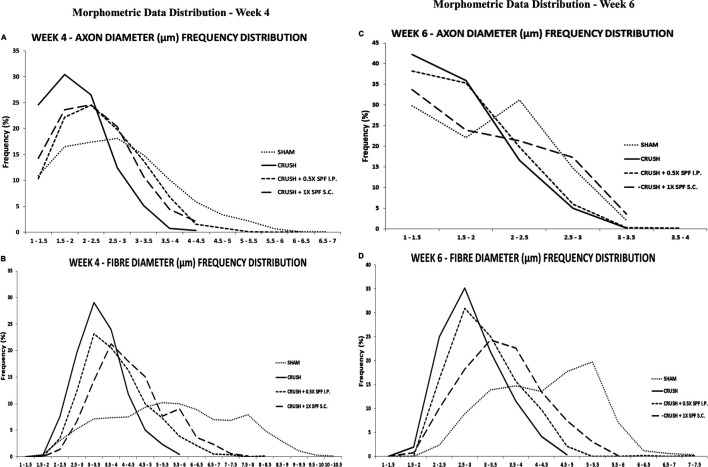
The percentage frequency of the myelinated axons (**A,C**) and nerve fibers (**B,D**) (*y*-axis) were plotted against the diameter sizes (*x*-axis) ranging from 1 µm to 10.5 µm from the experimental. The CRUSH group displayed a remarkable shift in the distribution of the axon and nerve fiber diameter sizes to the left toward the low range values compared to SHAM animals at week 4 and week 6. Although the treated groups showed an improvement pattern of the axon and nerve fiber diameters distribution, the CRUSH + SPF-FB(IP)-treated group displayed more normalization of axon and nerve fiber diameter distribution compared to the CRUSH + SPF-FB(IP)-treated group at Week 4 and Week 6.

Stereological calculation of myelinated axon diameters (d) (Figure 5D), myelinated nerve fiber diameter (D) (Figure 5E), and myelin thickness (Figure 5F) were obtained from 10 different random samples. The CRUSH group showed a significant (*p* < 0.002–*p* < 0.03) reduction in axon diameter at all the time points assessed compared with SHAM and CRUSH + SPF-FB-treated groups. However, IP and SC. SPF-FB-treated groups showed no significant change compared with SHAM animals at Week 4 and Week 6 post-nerve injury (Figure 5D). The nerve fiber diameter sizes (D) in the CRUSH and IP and SC. SPF-FB-treated groups at Week 4 and 6 displayed a significant (*p* < 0.0001) decrease compared to the SHAM group (Figure 5E). However, the nerve fiber diameter sizes in the CRUSH animals were significantly less compared to CRUSH + 0.5X SPF-FB(IP) at Week 4 (*p* < 0.001) and Week 6 (*p* < 0.04) following nerve injury. Likewise, the calculated myelin thickness for the CRUSH and CRUSH + SPF-FB-treated groups showed a significant (*p* < 0.001) decrease in myelin thickness compared to SHAM animals at Week 4 and Week 6 following sciatic nerve injury. However, the myelin thickness in CRUSH + SPF-FB-treated animals increased significantly (*p* < 0.003 - *p* < 0.05) at Week 4 and increased more at Week 6 compared with the CRUSH group. Western blot analysis of myelin basic protein (MBP) in the sciatic nerves is demonstrated in Figure 5G. The distal nerve samples’ MBP density analysis showed significantly increased basic myelin content in the IP and SC in support of the stereological analysis. SPF-FB-treated groups at Week 4 (*p* < 0.0001) and Week 6 (*p* < 0.01) post sciatic nerve injury. However, the MBP content increased in the IP SPF-FB-treated group did not reach the normal levels at Week 6 post-injury (Figure 5H). Analysis of the g-ratios (Table 1) revealed that the IP and SC. SPF-FB treatment significantly (*p* ≤ 0.0001 and *p* ≤ 0.01, respectively) lowered the axon diameter to myelinated fiber diameter ratio compared to the CRUSH group at Week 4 and Week 6 post-injury before normalization with control values. However, the g-ratio value of SC. SPF-FB treated group was still higher compared to sham and IP SPF-FB treated groups (Table 1). The g-ratio is calculated by the ratio of the axon to fiber diameter. Therefore, a higher g-ratio is expected in demyelinating neuropathy because remyelination leads to thinner myelin sheaths while the axon diameter remains unchanged. The decrease in the axon diameter to myelinated fiber diameter ratio may result from the presence of newly produced fibers with a thin myelin sheath in injured nerves leading to greater g-ratios. Otherwise, this decrease may be due to the loss of small myelinated nerve fibers since small myelinated nerve fibers produce higher g-ratios compared to large myelinated nerve fibers. The g-ratio examination is concurring with earlier studies that confirmed the efficacy of the SPF-FB treatment in speeding up the repair mechanism in the crush-injured nerves (Renno et al., 2016; Renno et al., 2017). The diameters of myelinated nerve axons and fibers were measured for additional confirmation of the morphometric data in Week 4 (Figures 6A,B) and Week 6 (Figures 6C,D) samples and the diameter distribution plotted. The CRUSH group displayed a remarkable shift in the distribution of the axon and nerve fiber diameter sizes to the left toward the low range values compared to SHAM animals at Week 4 and week 6. Although the treated groups showed an improvement distribution pattern of the axon and nerve fiber diameters, the CRUSH + SPF-FB(IP)-treated group displayed more normalization of axon and nerve fiber diameter distribution compared to the CRUSH + SPF-FB(SC)-treated group at Week 4 and Week 6 (Figure 6). To summarize, the morphometric data analysis of sciatic nerves crush injury treated with SPF-FB produced a significant positive effect on the remyelination of axons.

**TABLE 1:** G-ratio was determined as the ratio of the axon diameter to the fiber diameter.

Groups	Week 4	Week 6
SHAM	0.66238 ± 0.015297	0.65196 ± 0.002019
CRUSH	0.87582 ± 0.014100*	0.88024 ± 0.003148*
CRUSH + SPF-FB S.C.	0.76412 ± 0.023706**	0.74725 ± 0.012442**
CRUSH + SPF-FB IP	0.71928 ± 0.018457	0.64775 ± 0.009395

*p* ≤ 0.0001 Crush group compared to all groups; **p ≤ 0.01 Crush + SPF-FB S.C. compared to Sham and Crush + SPF-FB IP; values represent mean + SD.

### Histological and Immunohistochemical Analysis of Lumbar Spinal Cord

**FIGURE 7:**
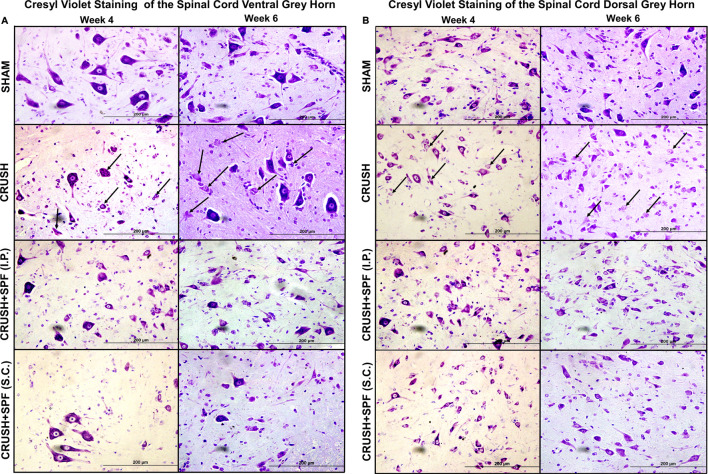
Photomicrographs of lumbar spinal cord **(A)**: ventral and **(B)**: dorsal grey horns from rats of all groups stained with Cresyl violet stain at Week 4 and Week 6 following sciatic nerve injury (100×). The degenerating neurons have prominent shrunken eosinophilic cytoplasm and are primarily characterized by nuclear pyknosis (either pyknotic or karyorrhectic nuclei). It is this heterogeneous appearance that typifies neuronal degeneration. Note fewer neurons and degenerating neurons (arrows) with less Nissl substance that appear pale because of the dissociation of ribosomes from the rough endoplasmic reticulum in the nerve-injured CRUSH than the SHAM group. The number of normally looking neurons is remarkably more in CRUSHvSPF-FB(IP) and CRUSH + SPF-FB(S.C.)-treated groups than the CRUSH group.

Histological examination of the Cresyl violet stained sections of the lumbar spinal cord ventral and dorsal grey horns at Week 4 and 6 after nerve injury are demonstrated in Figures 7A,B, respectively. The CRUSH animals revealed fewer healthy ventral and dorsal horn neurons and many degenerating neurons at Week 4 and Week 6 compared to the SHAM group. The number of neurons in the ventral and dorsal horns is remarkably more in CRUSH + SPF-FB(IP) group compared to the CRUSH group at Week 4 and Week 6.

**FIGURE 8:**
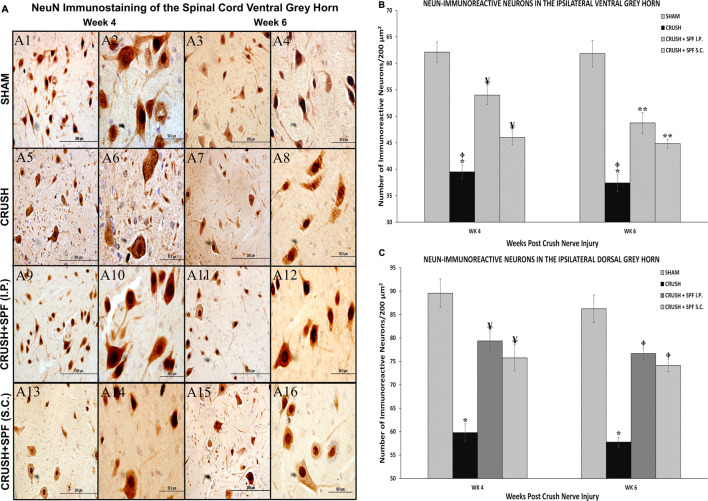
**(A)**: Representative 40× and 100× photomicrographs of lumbar spinal cord ventral grey horn immunostained for NeuN at Week 4 and Week 6 post-injury. Note less number of NeuN-immunostained neurons in the sciatic nerve-injured group (CRUSH) compared to the SHAM group. The number of the NeuN-immunoreactive neurons are remarkably more in the CRUSH + SPF-FB(IP) and CRUSH + SPF-FB (S.C.)-treated groups compared to the CRUSH group. **(B):** Bar graph shows the number of NeuN-immunoreactive neurons in the ipsilateral ventral grey horn at Week 4 and Week 6 post-injury. Note the significant decrease in the number of NeuN-immunostained neurons in the CRUSH group. Further, the number of neurons significantly increased in the CRUSH + SPF-FB (IP) and CRUSH + SPF-FB(S.C.)-treated groups at Week 4 and Week 6 post-injury. * indicates *p* < 0.0001 CRUSH vs. all groups; ¥ indicates *p* < 0.003 CRUSH + SPF-FB(IP) and CRUSH + SPF-FB(S.C.) vs. SHAM; Φ indicates *p* < 0.008 CRUSH vs. CRUSH + SPF-FB(S.C.); ** indicates *p* < 0.0001 CRUSH + SPF-FB(IP) and CRUSH + SPF-FB (S.C.) vs. SHAM. **(C)**: Bar graph shows the number of NeuN-immunoreactive neurons in the ipsilateral dorsal grey horn at week 4 and week 6 post-injury. Similarly, note the significant decrease in the number of neurons in the CRUSH group. In contrast, IP and S.C. SPF-FB treatments significantly increased the number of NeuN-immunoreactive neurons compared to CRUSH animals. * indicates *p* < 0.0001 CRUSH vs. All groups; ¥ indicates *p* < 0.02 CRUSH + SPF-FB(IP) and CRUSH + SPF-FB(S.C.) vs. SHAM; Φ indicates *p* < 0.004 CRUSH + 0.5X SPF-FB(IP) and CRUSH + 1X SPF-FB(S.C.) vs. SHAM.

NeuN-immunostaining (Supplementary Figure S4; low mag) of the ventral (Figure 8) and dorsal (Supplementary Figure S5) horns neurons of spinal cords showed fewer ventral and dorsal horn neurons in the CRUSH group at Week 4 (Figure 8A3) and Week 6 (Figure 8A4) compared to the SHAM group (Figures 8A1,A2, respectively). IP and SC. SPF-FB-treated groups showed more neurons compared to the CRUSH group at Week 4 (Figures 8A5,A6) and Week 6 (Figures 8A7,A8). The sciatic crush injured animals displayed a notable decrease in the number of NeuN-immunostained neurons of the ventral (Figure 8) and dorsal (Supplementary Figure S4) at Week 4 (Figures 8A5,A6) and Week 6 (Figures 8A7,A6) compared to SHAM group (Figures 8A1–A4). The number of the NeuN-immunoreactive neurons is remarkably more in the CRUSH + SPF-FB(IP) and CRUSH + SPF-FB (SC)-treated groups at Week 4 (Figures 8A9,A10 A13, respectively) and Week 6 (Figures 8A11,A12,A15,A16, respectively) compared to CRUSH group. Morphometric analysis of the ipsilateral ventral (Figure 8B) and dorsal (Figure 8C) grey horns showed a significant (*p* < 0.0001) decrease in the number of the NeuN-immunostained neurons in the CRUSH group compared to SHAM at Week 4 and Week 6. Further, the number of NeuN positive neurons significantly (*p* < 0.0001 and *p* < 0.008) increased in the CRUSH + SPF-FB (IP) and CRUSH + SPF-FB (SC)-treated groups at Week 4 and Week 6. In contrast, the number of NeuN-immunostained neurons significantly (*p* < 0.0001) increased in the CRUSH + SPF-FB (IP) and CRUSH + SPF-FB (SC)-treated groups at Week 4 and Week 6. However, the number of NeuN-immunostained neurons in the treated groups still significantly lower at Week 4 (*p* < 0.02) and Week 6 (*p* < 0.004) compared to SHAM animals. To summarize, SPF-FB treatment protected the spinal lumber neurons following sciatic nerve crush injury.

**FIGURE 9:**
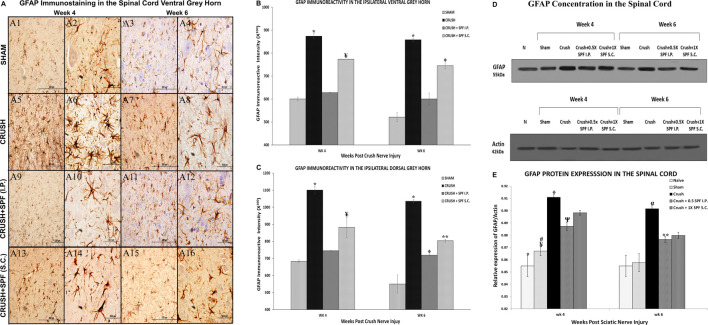
**(A)**: Representative 40× and 100× photomicrographs of lumbar spinal cord ventral grey horn immunostained for GFAP at Week 4 and Week 6 post-injury. Note an increasing number of GFAP-immunoreactive astrocytes in the CRUSH group compared to the SHAM and IP and S.C. SPF-FB-treated groups. **(B)**: Bar graph shows the number of GFAP-immunoreactive astrocytes in the ipsilateral ventral grey horn at week 4 and week 6 post-injury. Note the significant increase in the number of GFAP-immunoreactive astrocytes in the CRUSH group. In contrast, the number of GFAP-immunoreactive astrocytes significantly decreased in the CRUSH + 1X SPF-FB(S.C.) and CRUSH + 0.5X SPF-FB(S.C.)-treated groups. * indicates *p* < 0.0001 CRUSH vs. All groups; ¥ indicates *p* < 0.0001 CRUSH + 1X SPF-FB(S.C.) vs. all groups; Φ indicates *p* < 0.002 CRUSH + 0.5X SPF-FB(S.C.) vs. CRUSH and CRUSH + SPF-FB (IP). **(C)**: Bar graph shows the number of GFAP-immunoreactive astrocytes in the ipsilateral dorsal grey horn at week 4 and week 6 post-injury. Note the significant increase in GFAP-immunoreactive astrocytes in the CRUSH group compared to all experimental groups. Moreover, IP and S.C. SPF-FB treatments significantly decrease the number of GFAP-immunoreactive astrocytes at Week 4 and Week 6 following sciatic nerve injury. * indicates *p* < 0.0001 CRUSH vs. All groups; ¥ indicates *p* < 0.02 CRUSH + 1X SPF-FB(S.C.) vs. SHAM; Φ indicates *p* < 0.01 CRUSH + 0.5X SPF-FB(IP) vs. SHAM; ** indicates *p* < 0.001 CRUSH + 1X SPF-FB(S.C.) vs. SHAM. **(D)**: Western blot analysis of GFAP in the lumbar spinal cords at Week 4 and Week 6 following nerve injury. **(E)**: Bar graph shows the GFAP density in spinal cord samples. Note significantly decreased GFAP content in the CRUSH + 0.5X SPF-FB-FB (IP) spinal cords-treated groups. * indicates *p* < 0.0001 NAÏVE vs. All groups; ¥ indicates *p* < 0.0001 SHAM vs. CRUSH; # indicates *p* < 0.004 SHAM vs. CRUSH + 01X SPF-FB(S.C.) and CRUSH + 2X SPF-FB(S.C.); Φ indicates *p* < 0.002 CRUSH vs. CRUSH + SPF-FB(IP); Ψ indicates *p* < 0.003 SHAM vs. CRUSH + 0.5X SPF-FB(IP); α indicates *p* < 0.0001 CRUSH vs. NAÏVE and SHAM; ** indicates *p* < 0.05 CRUSH + 0.05X SPF-FB(IP) vs. CRUSH.

GFAP immunostaining showed a remarkable increase in the GFAP-immunoreactive astrocytes in the spinal cord (Figure 9A, Supplementary Figures S6, S7) in the CRUSH animals at Week 4 and Week 6 compared to the SHAM group (Figures 9A7,A8). The IP and SC. SPF-FB treatments resulted in an exceptionally low GFAP immunoreactivity in the ventral grey horn at Week 4 (Figures 9A9,A10,A13,A14, respectively) and Week 6 (Figures 9A11,A12,A15,A16, respectively) compared to CRUSH group. Likewise, the number of GFAP immunoreactive astrocytes in the dorsal grey horn is remarkably lower in the CRUSH + SPF-FB (IP) and CRUSH + SPF-FB (SC)-treated groups at Week 4 and Week 6 compared to the CRUSH group (See Supplementary Figure S7). Morphometric analysis of the ipsilateral ventral and dorsal grey horns showed a significant (*p* < 0.0001) increase in the number of the GFAP-immunostained astrocytes in the CRUSH group compared to SHAM at Week 4 and Week 6 post-injury (Figures 9B,C). Further, the number of GFAP positive astrocytes significantly (*p* < 0.0001) decreased in the CRUSH + SPF-FB (IP) treated groups at Week 4 and Week 6. However, the decrease of GFAP immunoreactivity in the CRUSH + SPF-FB (SC)-treated group remained significantly higher than the SHAM and CRUSH + SPF-FB (IP) groups at Week 4 (*p* < 0.0001) and Week 6 (ventral horn *p* < 0.002; dorsal horn *p* < 0.02). At week 6 post-injury, the IP and SC. SPF-FB-treated groups displayed significantly (*p* < 0.01 and *p* < 0.001) higher GFAP immunoreactivity in the dorsal grey horn compared to the SHAM group (Figure 9C). In support of the GFAP immunohistochemical staining and morphometric analysis, GFAPprotein density analysis lumbar of the spinal cord (Figures 9D,E) revealed a significant (*p* < 0.0001) increase in GFAP content in the CRUSH and SPF-FB-treated groups at Week 4 and Week 6 (Figure 9E). However, the SPF-FB-treated groups displayed a significant decrease in the GFAP protein concentration at Week 4 (*p* < 0.002) and Week 6 (*p* < 0.003) compared to the CRUSH group. Further, the GFAP protein content in the SPF-FB-treated animals was not significantly different from the SHAM group compared to NAÏVE and SHAM groups at Week 4 and Week 6 post-nerve injury in the spinal cord. In short, SPF-FB treatment decreased the number of GFAP-immunostained astrocytes in the spinal cord of the sciatic nerve crush injury animals.

**FIGURE 10:**
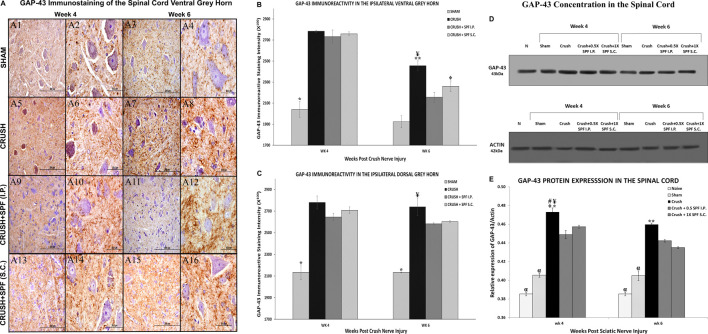
**(A)**: Representative 40× and 100× photomicrographs of lumbar spinal cord ventral grey horn immunostained for GAP-43 protein at week 4 and week 6 post-injury. Note the increase in the intensity of GAP-43 immunoreactivity in the CRUSH compared to all experimental groups. The GAP-43 immunoreactivity number is remarkably less in the CRUSH + SPF-FB(IP) and CRUSH + SPF-FB(S.C.)-treated animals compared to the saline-treated group. **(B)**: Bar graph shows the GAP-43 immunoreactive intensity in the ipsilateral ventral grey horn at week 4 and week 6 post-injury. Note significantly increased staining intensity in the CRUSH, CRUSH + 1X SPF-FB(IP), and CRUSH + 1X SPF-FB(S.C.) groups following nerve injury at Week 4. Whereas, IP and S.C SPF treatments significantly decreased the GAP-43 immunoreactivity at Week 6 post-injury. * indicates *p* < 0.001 SHAM vs. All groups; ¥ indicates *p* < 0.002 CRUSH vs. SHAM; epresentative 40× and 100× photomicrographs of lumbar spinal cord indicates *p* < 0.01 CRUSH + 1X SPF-FB(S.C.) vs. SHAM; ** indicates *p* < 0.03 CRUSH vs. CRUSH + 1X SPF-FB(IP) and CRUSH + 1X SPF-FB(S.C.). **(C)**: Bar graph shows the intensity of the GAP-43 immunoreactivity in the ipsilateral dorsal grey horn at week 4 and week 6 post-injury. Note significantly increased staining intensity in the CRUSH, CRUSH + 1X SPF-FB(IP) and CRUSH + 1X SPF-FB(S.C.) groups following nerve injury at Week 4. Whereas, IP SPF-FB treatment significantly decreased the GAP-43 immunoreactivity at Week 6 post-injury. * indicates *p* < 0.0001 SHAM vs. All groups; ¥ indicates *p* < 0.02 CRUSH vs. CRUSH + 0.5X SPF-FB(IP) and CRUSH + 1X SPF-FB(S.C.). **(D)**: Western blot analysis of GAP-43 in the spinal cord at Week 4 and Week 6 following nerve injury. **(E)**: Bar graph shows significantly increased GAP-43 content in the CRUSH, CRUSH + SPF-FB-treated groups compared to NAÏVE and SHAM groups at Week 4 and Week 6 post-injury. However, IP and S.C. SPF-FB treatments significantly decreased the GAP-43 protein content in the spinal cord compared to CRUSH animals. * indicates *p* < 0.0001 CRUSH vs. NAÏVE and SHAM; ¥ indicates *p* < 0.001 CRUSH vs. 0.5 CRUSH + 0.5X SPF-FB(IP); # indicates *p* < 0.02 CRUSH vs. CRUSH + 1X SPF-FB (S.C.); epresentative 40× and 100× photomicrographs of lumbar spinal cord indicates *p* < 0.009 CRUSH vs. CRUSH + 2X SPF-FB(S.C.); α indicates *p* < 0.0001 NAÏVE and SHAM vs. All groups; ** indicates *p* < 0.048 CRUSH vs. CRUSH + 0.5X SPF-FB(IP).

Growth-associated protein-43 (GAP-43) immunostaining in the lumbar spinal cord (Supplementary Figure S8) showed a general increase in the immunoreactivity in the CRUSH and SPF-FB-treated groups at Week 4 and Week 6 compared to the SHAM group. However, only at Week 6, the IP SPF-FB treatment decreased the GAP-43 immunoreactivity compared to the CRUSH group. The GAP-43 immunoreactivity in the ventral (Figure 10A) and dorsal (Supplementary Figure S9) grey horns of the CRUSH animals at Week 4 and Week 6 was remarkably higher compared to the SHAM group (Figures 10BA1–A4). The IP and SC. SPF-FB treatments resulted in a remarkably lower GAP-43 immunoreactivity in the ventral (Figures 10A11–A12, A15–A16, respectively) and dorsal (Supplementary Figure S9) grey horns at Week 6 compared to the CRUSH group. However, at Week 4 post-injury, the IP and SC. SPF-FB treatments did not reveal a noticeable difference compared to the CRUSH group (Figures 10 A9–A10, A13–A14, respectively). Staining intensity analysis of the ipsilateral ventral (Figure 10B) and dorsal (Figure 10C) grey horns showed a significant (*p* < 0.001 and 0.0001, respectively) increase in the GAP-43-immunostaining in the CRUSH and IP and SC. SPF-FB treated groups compared to SHAM at Week 4 and Week 6. The IP and SC. SPF-FB treatments decreased the GFAP immunoreactivity significantly (ventral horn *p* < 0.03; dorsal horn *p* < 0.02) compared to CRUSH group at Week 6. However, the decrease of the GAP immunoreactivity in the CRUSH + SPF-FB (SC)-treated group remained significantly (*p* < 0.01) higher than the SHAM group at Week 6. In support of the GAP-43 immunohistochemical staining and immunostaining intensity analysis, protein density examination of the spinal cord (Figures 10D,E) revealed a significant (*p* < 0.0001) increase in GAP-43 content in the CRUSH and SPF-FB-treated groups at Week 4 and Week 6 following sciatic nerve injury compared to NAÏVE and SHAM groups (Figure 10E). However, the SPF-FB-treated groups showed a significant (IP: *p* < 0.001, SC: *p* < 0.02) reduction in the GAP-43 protein content compared to CRUSH animals at Week 4. At Week 6 post-injury, the GAP-43 protein concentration in the SPF-FB-treated groups remained significantly (*p* < 0.04) lower than the CRUSH group. In short, SPF-FB treatment significantly decreased the GAP-43 protein content in the spinal cord of the crush nerve-injured animals, but not to control levels, signifying regenerating axons.

## Discussion

This study investigated several functional and biochemical parameters to address the effect of SPF-FB of PCEGS treatment on peripheral nerve regeneration, neural protection, and functional recovery following peripheral nerve crush injury. Our data showed that SPF-FB administration intraperitoneally or subcutaneously significantly improved the performance of different sensory and motor neurobehavioral functional tests in animals with nerve injury compared to the saline-treated control group. The SPF-FB treatments resulted in a substantial recovery in foot position and toe-spread analyses following injury. Likewise, SPF-FB-treated animals exhibited improvements in motor recovery measured by the extensor postural thrust, hopping response, and Rotarod. Also, the tactile allodynia and plantar mechanical hyperalgesia thresholds significantly decreased compared to the CRUSH group. Nerve crush injury produced severe nociception deficits in both heat withdrawal reflex and tail-flick withdrawal latency tests, which were markedly recuperated in SPF-FB-treated animals. The neurobehavioral data were supported by significant histomorphological evidence of axonal regeneration following SPF-FB treatments compared to controls at Weeks 4 and 6 postoperatively.

Further, the CRUSH group displayed a significant increase in the total small and thinly myelinated axon number at weeks 4 and 6 post-injury significantly reduced following SPF-FB treatment indicating constructive regeneration axonal recovery. Likewise, SPF-FB treatment increased the axon area, average axon perimeters, and myelin thickness and decreased g-ratio. Further, the SPF-FB treatments remarkably ameliorated the neurodegenerative changes seen in ventral and dorsal grey horns of the spinal cord sections ipsilateral to the nerve injury and significantly prevented the decrease in the number of spinal neurons with a concomitant reduction in the astrocytes and the GAP-43 immunoreactivity.

In this study, the neuroprotective effect of the SPF-FB in treating the nerve injury agrees with previous studies, which showed the potency of the healing and anti-inflammatory properties of some components in the preparations. The biochemically and pharmacologically active components of PCEGS are effective in a high percentage of the 75 cases of diabetic foot ulcers and 94 cases of different neurological disorders. PCEGS displayed potent anti-inflammatory and healing properties, as shown in early clinical trials, to heal non-healing diabetic foot ulcers, chronic back pain, and other neurological disorders (Al-Hassan 1990; Al-Hassan et al., 1991; Al-Hassan et al., 2005; Al-Hassan et al., 2006; Al-Hassan et al., 2007; Al-Hassan et al., 2020). These preliminary data have indicated that PCEGS had a noticeable effect on neuropathy in the diabetic foot and potent healing effects on the chronic back and joint pain (Al-Hassan 1990; Al-Hassan et al., 1991; Al-Hassan et al., 2005; Al-Hassan et al., 2006; Al-Hassan et al., 2007; Al-Hassan et al., 2020). Further, PCEGS treatment completely healed the gangrenous feet, resulted in the natural degradation of the necrotic tissues, and some sensation returned to the neuropathic extremity of patients with non-healing foot ulcers or wet gangrene ulcers (Al-Hassan 1990). The growth rate of new tissues was proportional to the amount of healing material applied, and its effect ceased shortly after the interruption of the treatment. No atrophy of the skin lesions was noted, and no side-effects were detected (Al-Hassan 1990; Al-Hassan et al., 2020).

Before the process of regeneration can occur in the damaged nerve, a sequence of degenerative processes must occur depending on the type and severity of the injury, whether motor or sensory neurons and myelinated or unmyelinated fibers are injured, and on consequences such as deficits in axonal transport or demyelination (Mietto et al., 2015). Axonal degeneration is most likely to be triggered distal to the crush injury site combined with specific histological changes at the injury site or proximal to it (Mietto et al., 2015). In an anterograde manner, both axon and surrounding myelin breakdown until they reach the distal part of the nerve segment. Both the neurotubules and neurofilaments become disarrayed due to axonal swelling leading to fragmentation of the axon. Within 24 h of injury, Schwann cells (SCs) become active and divide rapidly to give differentiated daughter cells that downregulate myelin-specific proteins as well as upregulate several glial growth factors (Burnett and Zager, 2004). With the help of the invading macrophages, SCs start removing the degenerated axonal and myelin debris.

Meanwhile, endoneurial mast cells release histamine and serotonin to increase capillary permeability and expedite macrophages’ migration through the capillaries. The primary role of axonal degeneration is to remove axon and myelin-derived material to pave the way for the axons to regenerate within 5–8 weeks (Burnett and Zager, 2004). Consequently, the first 2 weeks are the most crucial period of the neurodegeneration process, where inflammation, degeneration of myelinated axons, removal of the dead myelin, and neuronal SCs damage processes occur. Thus, it is imperative to intervene within this time frame to influence these processes and protect SCs in the sciatic nerve and the spinal cord’s neurons from the apoptotic processes. In our study, the SPF-FB treatments started immediately after the induction of the nerve injury. They lasted for almost two weeks to lessen the effect of secondary inflammation and degenerative processes while boosting the process of regeneration, which starts at the end of the second week following the crush injury. These histological results are consistent with our previous studies (Thalhammer et al., 1995; Luis et al., 2007; Renno et al., 2013; Renno et al., 2017).

Examination of SPF-FB-treated nerve sections revealed a remarkable increase in the number of newly regenerated nerve fibers enclosed with more myelin compared to CRUSH nerves. The SPF-FB-treated nerves showed approximately similar normal morphology to NAÏVE by the end of the 6th-week post-surgery compared to CRUSH nerves. Many nerve fibers with normal histology and small, thinly ensheathed myelin may be designated as regenerated nerve fibers still in the process of remyelination. On the contrary, the crushed nerves showed healthy unmyelinated nerve fiber bundles that may be identified as either regenerated unmyelinated axonal fibers or newly regenerated axonal fibers with incomplete remyelination processes (Varejão et al., 2004; Varejão et al., 2004; Luis et al., 2007; Renno et al., 2013; Renno et al., 2017; Hussain et al., 2018). The increase in MBP protein concentration biochemically confirmed the histologically observed increase in the SPF-FB-treated nerves’ myelin.

In this study, SPF-FB neurobehavioral recovery and the morphological improvements in the histological architecture of the crush-injured nerves were supported by the increased survival of neurons in the ventral and dorsal horns of spinal cords of SPF-FB-treated rats. In line with our previous studies, the CRUSH group showed a significant decrease in the number of the motor and sensory neurons at the L4-L5 spinal cord levels ipsilateral to the injury site compared to the NAÏVE and SHAM groups (Luis et al., 2007; Renno et al., 2013; Renno et al., 2017). After axonal damage, significant neuronal cell death and axon degeneration occur, leading to functional deficits (Luis et al., 2007; Renno et al., 2013; Renno et al., 2017). The number of neurons in the ipsilateral spinal cord horns was significantly more in SPF-FB-treated groups compared to the CRUSH group at Week 4 and Week 6 following the nerve crush injury. Also, the SPF-FB treatment reduced the number of astrocytes and GAP-43, a marker of regenerating axons. GFAP is the central intermediary filament for astrocytes and is a marker for reactive gliosis related to neuronal damage and aging (Rrapo et al., 2009; Middeldrop and Hol, 2011). In this study, the SPF-FB treatment significantly decreased the GFAP-immunoreactivity and protein levels in the spinal cord segments compared to the CRUSH group at Week 4 and Week 6. This neuroprotection is achieved by preventing gliosis and neuronal damage leading to the promotion of cellular regeneration. Astrocytes are vital for healthy CNS function, and alterations in their activity could lead to neuronal impairment (Coleman et al., 2010). Astrocytes usually play numerous roles in neuronal activity, including neurotransmitter release, regulation of ion flux currents, synaptogenesis, and energy production. Reactive astrogliosis may upsurge the production of various cytokines and chemokines such as TGF-α, IL-1α, IL-6, ciliary neurotrophic factor (CNTF). Also, adhesion/recognition molecules and proteins such as COX2, inducible nitric oxide (iNO) synthase, and calcium-binding protein S100a, which drive the secondary neuronal damage, may be increased (Pasterkamp et al., 2006).

Further, astrogliosis can exert both beneficial and detrimental effects depending on signaling pathways activated and timing after crush nerve injury. Astrogliosis minimizes secondary tissue damage by limiting the damaged area, providing growth factors, restoring blood-spinal cord barrier and tissue structure, revascularization, maintaining homeostasis, and removing tissue debris from the injured area (Detloff et al., 2008). However, recent studies revealed the importance of timing in modulating various aspects of reactive astrocytes after neuronal injury (Karimi-Abdolrezaee and Billakanti, 2012). Therefore, reactive astrocytes’ beneficial role is mainly at the early stages of injury or secondary inflammation process. As time passes following injury, reactive astrocytes’ inhibitory properties progress and overcome their constructive effects that are mainly accredited to the upregulation of inhibitory molecules that actively obstruct neural repair and regeneration (Karimi-Abdolrezaee and Billakanti, 2012). In this regard, our data showing an increase in the astrocytes following nerve crush injury agrees with their crucial initial role in containing the secondary damage and recruiting and manipulating the subsequent inflammatory response. It has been shown earlier that astrocytes increase in number and migrate to the site of injury (Schwab and Bartholdi, 1996). The decrease in the reactive astrogliosis following the SPF-FB treatment may indicate a tuning down of the inflammatory process and less recruitment of neutrophil and macrophages infiltration at the crush injury nerve site and the corresponding spinal cord segments leading to improved motor function, tissue sparing, and neuroprotective effects.

Our data are consistent with the notion proposed by Nesic and collaborators (Nesic et al., 2005) that inhibition of glial activation, both astrocytic and microglia, would improve chronic and persistent pain syndromes in remote segments below the level of the lesion after spin""Fal cord injury. This study demonstrated significant GFAP protein increases in concurrence with the rise in pain threshold measured by different hyperalgesia parameters in the crush injury animals. In contrast, SPF-FB treatments resulted in the reduction of central and peripheral neuropathic pain with a significant decrease in GFAP protein expression and “gliopathy.” The mechanisms of SPF-FB of the PCEGS reduction in “gliopathy,” leading to remarkable neurobehavioral recovery and neuronal protection, remained established.

Our study examined the effect of SPF-FB on the GAP-43, which is a membrane protein involved in neuronal development and plasticity and found in the growth cones and actively remodeling terminals. GAP-43 is thus used as a neuronal marker of regions undergoing neurite outgrowth or capable of plasticity in adult animals (Rekart et al., 2004). It is also considered a vital factor for proper neuronal regeneration (Rezai-Zadeh et al., 2005). The GAP-43 expression has been shown to increase and then transported peripherally following peripheral nerve damage, contributing to neuronal regeneration (Rezai-Zadeh et al., 2005). Our data agree with the role of the GAP-43 protein as an active remodeling factor in the regions undergoing neurite outgrowth for proper neuronal regeneration. The increase in GAP-43 in the CRUSH and CRUSH + SPF-FB-treated groups indicate that the process of axonal regeneration and neuronal outgrowth in the spinal cord is continuing at Week 4. The fact that the SPF-FB treatment significantly reduced GAP-43 content, although not to control levels, indicates that the need for GAP-43 decreases as neuroregeneration proceeds to its completion, which takes place approximately by Week 10 post nerve injury (Renno et al., 2013; Renno et al., 2017). The reduction in the GAP-43 levels seen in the SPF-FB-treated animals is in line with the Rekart and coworkers findings that the elevated levels of GAP-43 leading to potentially aberrant sprouting were positively correlated with the severity of Alzheimer’s Disease, suggests that the increased expression of GAP-43 leads to a miswiring of circuits critical for memory function (Rekart et al., 2004).

Although the use of 3 mg SPF-FB/kg animal body weight proved to be an excellent dose in the present neurological studies, in treating pancreatic cancer and in treating STZ-induced diabetes in animals, our future work will further explore the best dose. More work will be required to elaborate on the mechanism(s) of action of the SPF-FB. Our results show the novelty of our study. We could not compare our data with any other study since no animal work was done on the neuroprotective properties of SPF-FB on peripheral or central neuronal degenerative diseases.

Further experiments are also needed to confirm the neuroprotective effects in another model like the lipopolysaccharide (LPS) neuroinflammation model or other neurodegenerative disease animal models. It is well known that the inflammatory and degenerative processes in the nerve occur in the first 2 weeks following crush injury then subside to allow for the regenerative process to take place. Hence, short biochemical and molecular mechanistic studies need to be done in the first 2 weeks post-injury and after initiation of SPF-FB treatments. Our previous studies showed that PCEGS and its SPF-FB contain a complex mixture of biochemically and pharmacologically active lipids and proteins. Some lipid and protein components are needed together for the repair and regeneration processes and act in synergism. Some biologically active lipids, such as eicosanoids, steroids, and furan fatty acids, exist in minute amounts and can only be detected by Gas chromatography-Mass Spectrometer (GC-MS). The separation of the biologically active lipids into single components may lead to their oxidation and changes in their structures and the loss of their activities. Our biochemical analysis showed that SPF-FB encompasses several growth factors such as nerve growth factor (NGF), fibroblasts growth factors 1 (FGF1), 19 (FGF19), and 21 (FGF21), and Substance P (data not shown). Their involvement in the sequence of the repair and regeneration events has yet to be established.

## Conclusion

Our results support our hypothesis that SPF-FB treatment alleviates neurobehavioral deficits, stimulates the axons’ regeneration, and causes histomorphological alterations following nerve injury (Flow Chart). Further, fraction B of the soluble protein fraction B protects spinal neurons and enhances subcellular recovery after peripheral nerve injury, thus improving nerve regeneration. This study established the different therapeutic characteristics and the mechanisms through which SPF-FB could produce its therapeutic effects on the injured nerve. These results coupled with our previous study results (Wang et al., 2001), showed that, when a preparation from the catfish skin was applied topically to rat skin caused porosity of the blood vessels and diffusion of a dye under the skin. This help understand the SPF-FB diffusion process involved when it was applied subcutaneously for treatment of the crushed nerve. Also, it explains the therapeutic outcomes when SPF-FB was applied topically for the treatement of some human neurological diseases. As some neurological diseases are so far difficult or impossible to cure, we hope our results will raise scientific interest into employing Fraction B or components therefrom as therapeutic agents, or as part of repair strategies to cure nerve injury or some of these difficult to cure neurological diseases.

## Data Availability

The raw data supporting the conclusions of this article will be made available by the authors, without undue reservation.

## Abbreviations:

C-20 fatty acid, (12,15-epoxy-13,14-dimethyleicosa-12,14-dienoic acid, a furanoic acid or F-6); CNTF, ciliary neurotrophic factor; COX2, cyclooxygenase 2; EPT, extensor postural thrust; FGF1, fibroblasts growth factors 1; FGF19, fibroblasts growth factors 19; FGF21, fibroblasts growth factors 21; GAP-43, growth-associated protein-43; GC-MS, gas chromatography-mass spectrometry; GFAP, Glial fibrillary acidic protein; IL-1α, interlukin-1α; IL-6, inerlukin-6; iNO, inducible nitric oxide; IP, intraperitoneal; L4-L5, lumbar spinal cord segments 4 and 5; MBP, myelin basic protein; NeuN, NEUronal Nuclei; NGF, nerve growth factor; PAF, platelet-activating factors; PBS, phosphate buffer saline; PCEGS, Arabian Gulf catfish (*Arius bilineatus*, Val) epidermal gel secretion; PGF_2α_, prostaglandinF_2α_; SC, subcutaneous injection; SCs, schwann cells; SPF-FB, soluble protein fraction B; TGF-α tumor growth factor-α; WRL, withdrawal response latency
