# Supplementary material for: Catfish Epidermal Preparation Accelerates Healing of Damaged Nerve in a Sciatic Nerve Crush Injury Rat Model
Source: Front Pharmacol. 2021 Apr 14;12:632028. doi: 10.3389/fphar.2021.632028 (PMC8112254; doi:10.3389/fphar.2021.632028)
Supplement: Supplementary file 1 [file Image1.tif]

Frontiers | Catfish (Arius bilineatus, Val.) epidermal protein preparation ameliorates neurobehavioral and axonal and neuronal histopathological alterations in a sciatic nerve crush injury rat model. | Pharmacology


- About
- Journals
- Research Topics
- Articles
- More

Submit

My Frontiers

Office

- TSOF
  - TSOF
  - Article Production

Typesetter 3

frontiersproduction@tnq.co.in

- Profile
- Settings & Privacy
- Help Center
- Logout

Submit

**Impact Factor 4.225** | **CiteScore 5.0**More on impact ›

|  |  |
| --- | --- |
| Frontiers in Pharmacology | Ethnopharmacology |

Toggle navigation


Section


- (current)Section
- About
- Articles
- Research topics
- For authors 
  - Why submit?
  - Fees
  - Article types
  - Author guidelines
  - Review guidelines
  - Submission checklist
  - Contact editorial office
  - Submit your manuscript
- Editorial board

- *Article alerts*

##### This article is part of the Research Topic

Natural Bioactive Compounds and Neurodegenerative Disorders: From Molecular Mechanisms Towards Clinical Therapies
View all
3
Articles

Articles


**Suggest a Research Topic >**

- 107
  total views

 View Article Impact

**Suggest a Research Topic >**

##### SHARE ON

- Facebook

  0
- Twitter

  0
- LinkedIn

  0
- AddThis

  New


## Original Research ARTICLE

Front. Pharmacol.
| doi: 10.3389/fphar.2021.632028

# Catfish (Arius bilineatus, Val.) epidermal protein preparation ameliorates neurobehavioral and axonal and neuronal histopathological alterations in a sciatic nerve crush injury rat model. Provisionally accepted The final, formatted version of the article will be published soon. **Notify me**

Waleed M. Renno1\*,  Jassim M. Al-Hassan2, Mohammad Afzal2, 
 BINCY M. PAUL2,  Divya J. Nair2 and Jijin Kumar1

- 1Faculty of Medicine, Kuwait University, Kuwait
- 2Kuwait University, Kuwait

Preliminary investigations showed that preparations from Arabian Gulf catfish (Arius bilineatus, Val) epidermal gel secretion (PCEGS) exhibit potent anti-inflammatory and healing properties as shown in our previous clinical trials for the healing of non-healing diabetic foot ulcers, chronic back pain, and some other neurological disorders. Here, we report for the first time a unique preparation containing only proteins and lipids (soluble protein fraction B, SPF-FB), derived from the PCEGS accelerated the healing and recovery of sensory-motor functions of experimental sciatic nerve crush injury in rats with its unique neuroprotective and neuroregenerative properties on the spinal neurons and peripheral nerve fibers. Male rats were randomly assigned to Five groups: (I) NAÏVE, (II) SHAM, (III) CRUSH treated with saline, (IV) CRUSH+SPF-FB treated with 3 mg/kg intraperitoneally (IP), and (V) CRUSH+SPF-FB treated with 6 mg/kg subcutaneously (SC) groups. The crush groups III, IV, and V underwent sciatic nerve crush injury, followed by treatment daily for 14 days with saline, SPF-FB IP and SPF-FB SC. All animals were tested for the neurobehavioral parameters throughout the 6 weeks of the study. Sciatic nerve and spinal cord tissues were processed for light and electron histological examinations, stereological analysis, immunohistochemical and biochemical examinations at Week 4 and Week 6 post-injury. Administration of SPF-FB IP or SC significantly enhanced the neurobehavioral sensory and motor performance and histomorphological neuroregeneration of the sciatic nerve-injured rats. The stereological evaluation of the axon area, average axon perimeters, and myelin thickness revealed significant histomorphological evidence of neuroregeneration in the FB-treated sciatic nerve crush injured groups compared to controls at 4 and 6 weeks. SPF-FB treatment significantly prevented the decrease in NeuN-immunoreactive neurons, decreased GFAP immunoreactive astrocytes, and increased GAP-43. We conclude that SPF-FB treatment lessens neurobehavioral deficits, enhances axonal regeneration following nerve injury. Further, SPF-FB protects spinal neurons and enhances subcellular recovery by decreasing astrocytic activity and GAP-43, thus improves nerve regeneration and functional recovery. SPF-FB preparation of the PCEGS contains the components necessary to protect the degenerating spinal cord neurons and enhancing the regeneration of injured sciatic nerve fibers following crush injury.

Keywords: 
Neuroprotection, Neuroregeneration, Nerve injury, neurobehavioral, Catfish epidermal mucus extracts

Received: 21 Nov 2020;
Accepted: 18 Jan 2021.

Copyright: © 2021 Renno, Al-Hassan, Afzal, PAUL, Nair and Kumar. This is an open-access article distributed under the terms of the Creative Commons Attribution License (CC BY). The use, distribution or reproduction in other forums is permitted, provided the original author(s) and the copyright owner(s) are credited and that the original publication in this journal is cited, in accordance with accepted academic practice. No use, distribution or reproduction is permitted which does not comply with these terms.

\* Correspondence: 
Prof. Waleed M. Renno, Faculty of Medicine, Kuwait University, Kuwait City, 13060, Kuwait, waleed.renno@ku.edu.kw

Write a comment...

Add

##### COMMENTARY

##### ORIGINAL ARTICLE

##### People also looked at

## The Burden of the Serious and Difficult-to-Treat Infections and a New Antibiotic Available: Cefiderocol

Yasaman Taheri, Nataša Joković, Jelena Vitorović, Oliver Grundmann, Alfred Maroyi and Daniela Calina

## Catalpol Protects Against Spinal Cord Injury in Mice Through Regulating MicroRNA-142-Mediated HMGB1/TLR4/NF-κB Signaling Pathway

Hougang Xia, Dandan Wang, Xiaohui Guo, Kaidi Wu, Fuwei Huang and Yanjiang Feng

## Pharmacological Properties of Chalcones: A Review of Preclinical Including Molecular Mechanisms and Clinical Evidence

Bahare Salehi, Cristina Quispe, Imane Chamkhi, Nasreddine El Omari, Abdelaali Balahbib, Javad Sharifi-Rad, Abdelhakim Bouyahya, Muhammad Akram, Mehwish Iqbal, Anca Oana Docea, Constantin Caruntu, Gerardo Leyva-Gómez, Abhijit Dey, Miquel Martorell, Daniela Calina, Víctor López and Francisco Les

## The Species Identification in Traditional Herbal Patent Medicine, Wuhu San, Based on Shotgun Metabarcoding

Jinxin Liu, Weishan Mu, Mengmeng Shi, Qing Zhao, Weijun Kong, Hongbo Xie and Linchun Shi

## Mingmu Xiaomeng Tablets Restore Autophagy and Alleviate Diabetic Retinopathy by Inhibiting PI3K/Akt/mTOR Signaling

Yuwei Fang, Kangpei Shi, Haining Lu, Lin Lu and Bo Qiu

**Suggest a Research Topic >**

×

#### Supplementary Material

  

There is no supplementary material currently available for this article

Loading supplemental data...

  

|  | File Name |  |
| --- | --- | --- |
|  | Data Sheet 1.PDF |  |
|  | Image 1.TIF |  |

  

Close

- About Frontiers
- Institutional Membership
- Books
- News
- Frontiers' social media
- Contact
- Careers
- Submit
- Newsletter
- Help Center
- Terms & Conditions
- Privacy Policy

© 2007 - 2021 Frontiers Media S.A. All Rights Reserved

### Privacy Preference Center

Our website uses cookies that are necessary for its operation. Additional cookies are only used with your consent. These cookies are used to store and access information such as the characteristics of your device as well as certain personal data (IP address, navigation usage, geolocation data) and we process them to analyse the traffic on our website in order to provide you a better user experience, evaluate the efficiency of our communications and to personalise content to your interests. Some cookies are placed by third-party companies with which we work to deliver relevant ads on social media and the internet. Click on the different categories' headings to change your cookie preferences. Click on "More Information" if you wish to learn more about how data is collected and shared.
More information

### Manage Consent Preferences

#### Strictly Necessary Cookies

Always Active

These cookies are necessary for the website to function and cannot be switched off in our systems. They are usually only set in response to actions made by you which amount to a request for services, such as setting your privacy preferences, logging in or filling in forms. You can set your browser to block or alert you about these cookies, but some parts of the site will not then work. These cookies do not store any personally identifiable information.

#### Analytics Cookies

Analytics Cookies

These cookies allow us to count visits and traffic sources so we can measure and improve the performance of our site. They help us analyse which pages are the most and least popular and see how visitors move around the site.    All information these cookies collect is aggregated and therefore anonymous.

#### Functional Cookies

Functional Cookies

These cookies enable the website to provide enhanced functionality and personalisation. They may be set by us or by third party providers whose services we have added to our pages. If you do not allow these cookies then some or all of these services may not function properly.

#### Advertising Cookies

Advertising Cookies

These cookies may be set through our site by our advertising partners. They may be used by those companies to build a profile of your interests and show you relevant adverts on other sites.    They do not store directly personal information, but are based on uniquely identifying your browser and internet device. If you do not allow these cookies, you will experience less targeted advertising.

### Back Button Performance Cookies

Vendor Search  Search Icon

Filter Icon

Clear

checkbox label label

Apply Cancel

Consent Leg.Interest

checkbox label label

checkbox label label

checkbox label label

Confirm My Choices
