# Supplementary material for: Catfish Epidermal Preparation Accelerates Healing of Damaged Nerve in a Sciatic Nerve Crush Injury Rat Model
Source: Front Pharmacol. 2021 Apr 14;12:632028. doi: 10.3389/fphar.2021.632028 (PMC8112254; doi:10.3389/fphar.2021.632028)
Supplement: Supplementary file 2 [file datasheet1.pdf]

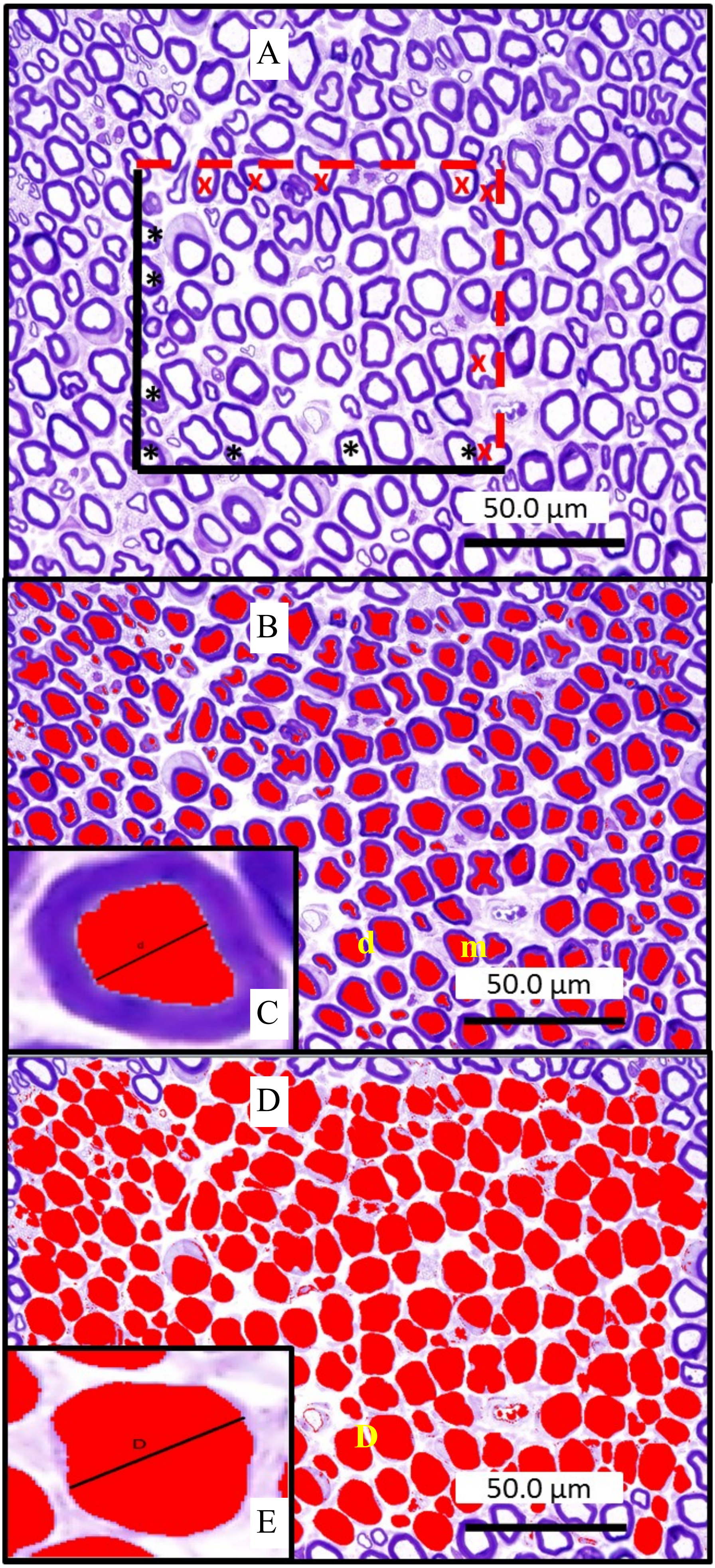

Suppl Fig. 1 SA: Representative photomicrograph of a cross-section of sciatic nerve myelinated axons stained with toluidine blue. The unbiased counting frame method with a square sample area was used to count the myelinated axons. The nerve profiles marked with yellow asterisks along the solid yellow lines (inclusion lines) were included in the counts, but the profiles marked with red Xs touching the broken red lines (exclusion lines) were excluded. **B–E:** Representative photomicrographs of a cross-section of sciatic nerve myelinated axons stained with toluidine blue demonstrating the method of calculating myelin thickness and g-ratios. For calculating the myelin thickness (m = [D-d]/2) and g-ratio (d/D), the axon diameter (d) and the nerve fiber diameter (D) were measured automatically using Image-Pro Plus 6.0. Thickness measurements from all sampled axons were averaged to obtain the mean myelin thickness.

Suppl Fig. S2: Toluidine blue stained photomicrographs of semi-thin transverse sections of sciatic nerves obtained from animals in the SHAM (**A, B and C**), CRUSH (**D, E and F**), CRUSH+0.5X SPF(I.P.) (**G, H and I**) and CRUSH+1X SPF(S.C.) (**J, K and L**) groups at ***Week 4*** following nerve injury (Column I; 10x, Column II, 40x and Column III, 100x). Sciatic nerves from CRUSH (**D, E and F**) animals show the presence of smaller mini-fascicles nerve fibers with less myelin and macrophages filled with degraded myelin (arrows) following crush injury. In contrast, sciatic nerve sections of CRUSH+0.5X SPF(I.P.) (**G, H and I**) and CRUSH+1X SPF(S.C.) (**J, K and L**) groups showed remarkable nerve regeneration with large nerve fibers surrounded with noticeable increase in myelin layers compared with those from the CRUSH (**D, E and F**). Also, notice that myelin debris and macrophages in the SPF-treated groups were much less compared to CRUSH nerves.

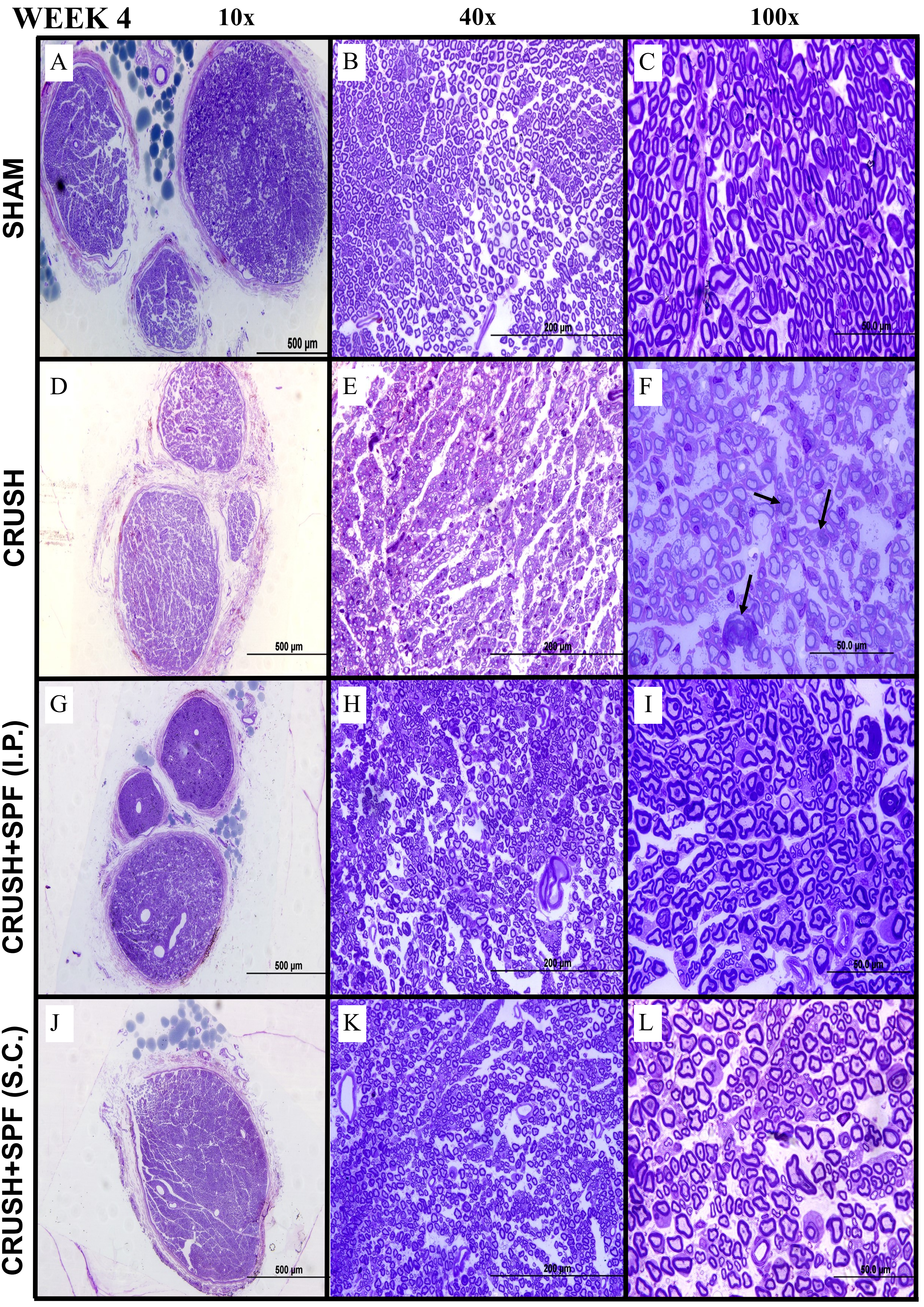
WEEK 4
10x
40x
100x
SHAM
CRUSH
CRUSH+SPF (I.P.)
CRUSH+SPF (S.C.)
A
B
C
D
E
F
G
H
I
J
K
L
500 µm
200 µm
50.0 µm

Suppl Fig. S3: Electron micrographs of sciatic nerve from the SHAM (**A and B**), CRUSH (**C and D**), and CRUSH+SPF(I.P.) (**E and F**) and CRUSH+SPF(S.C.) (**G and H**) groups at ***Week 4*** post-injury (Panel I; 5000x and Panel II; 10000x). Note very irregular shaped and highly condensed abnormal myelin sheaths (arrows) and axonal fibers along with disintegrated and remnants of myelin scattered in between the axons (asterisk) and a considerable number of unmyelinated or slightly myelinated axons (U) in the CRUSH group (I). SPF-treated crushed sciatic (**E, F, G and H**) nerves showed normal, healthy appearing myelin sheaths, with normal thickness and normal axons. Also, note the healthy normally appearing Schwann cell in SPF-treated animals. M-myelin sheath; AX- axons.

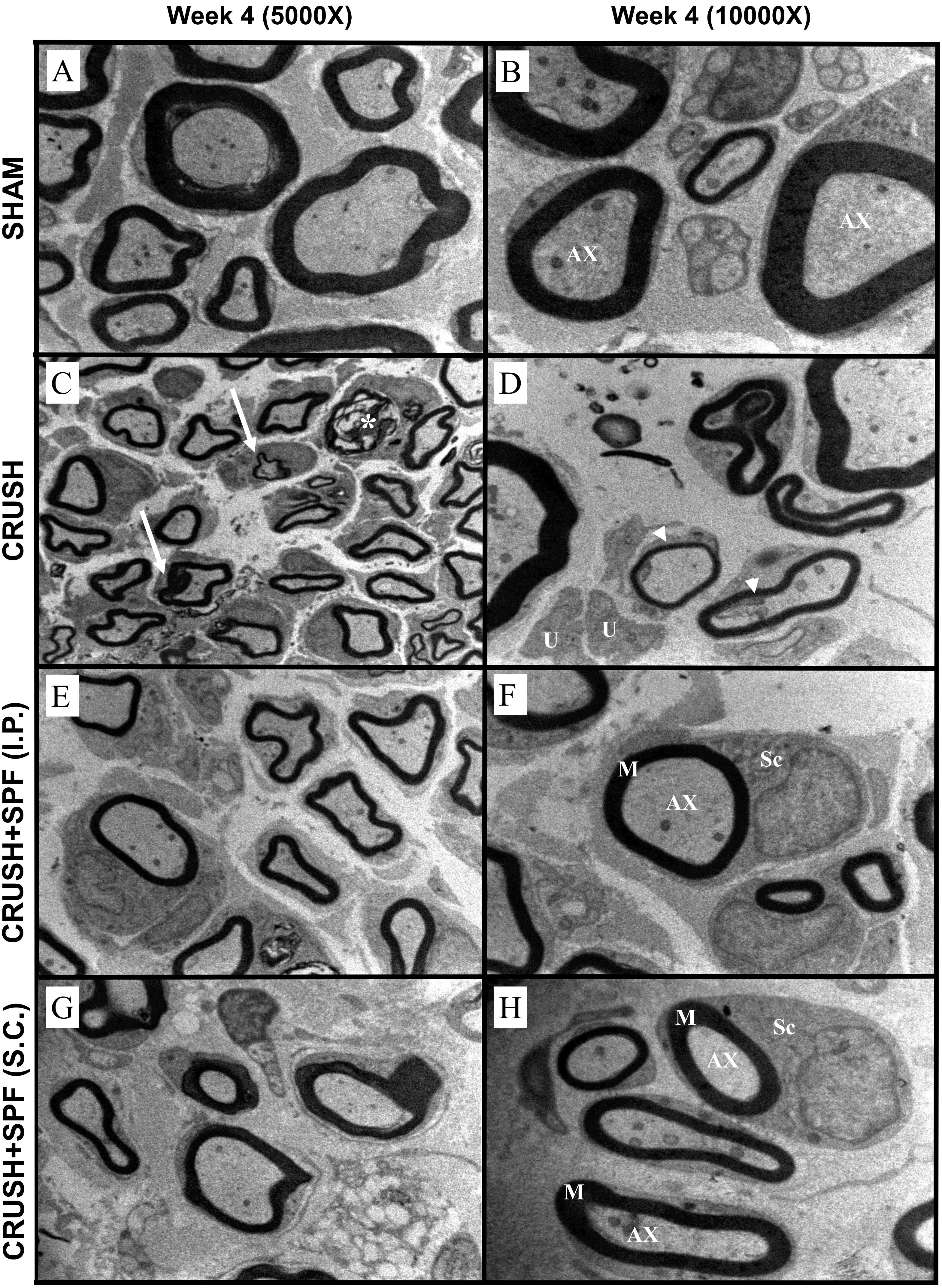
Week 4 (5000X)
Week 4 (10000X)
SHAM
CRUSH
CRUSH+SPF (I.P.)
CRUSH+SPF (S.C.)
A
B
AX
AX
C
*
D
U
U
E
F
M
Sc
AX
G
H
M
Sc
AX
M
AX

Suppl Fig. S4 Representative 10x photomicrographs of the lumbar spinal cord from rats of all groups immunostained for NeuN at Week 4 and Week 6 following nerve crush injury. Note less number of neurons in the CRUSH animals at Week 4 (A3) and Week 6 (A4) compared to the SHAM group (A1 and A2, respectively). The number of neurons being noticeably more in the CRUSH+SPF(I.P.) and CRUSH+SPF(S.C.)-treated groups at Week 4 and Week 6 compared to CRUSH group.

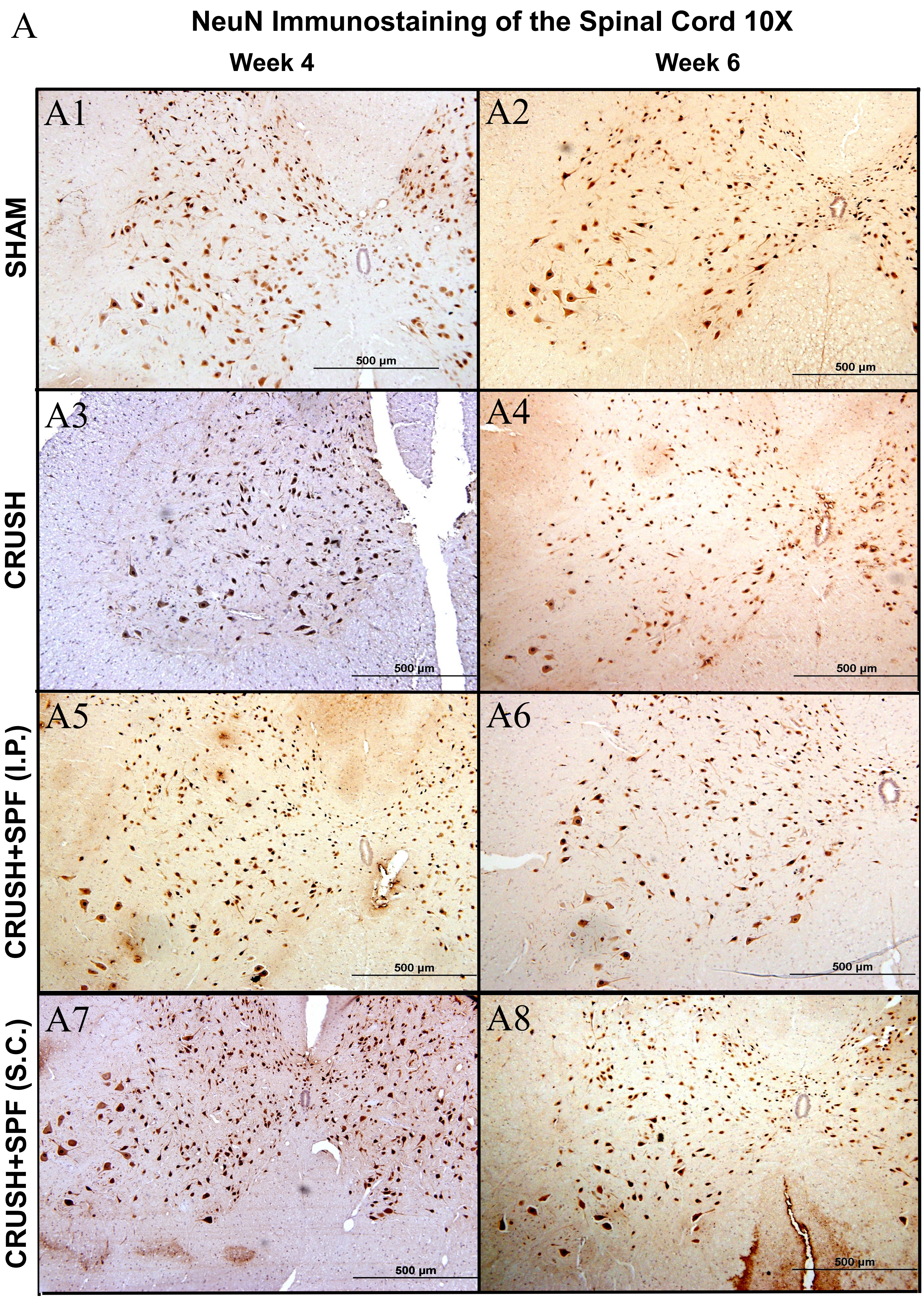
A
NeuN Immunostaining of the Spinal Cord 10X
Week 4
Week 6
SHAM
CRUSH
CRUSH+SPF (I.P.)
CRUSH+SPF (S.C.)
A1
A2
A3
A4
A5
A6
A7
A8
500 µm

Suppl Fig. S5 Representative 40x and 100x photomicrographs of lumbar spinal cord dorsal grey horn from rats of the experimental groups immunostained for NeuN at week 4 and week 6 post-injury. Note less number of neurons and degenerating neurons (arrows) in the sciatic nerve-injured group (CRUSH+SALINE) compared to NAÏVE and SHAM groups. The number of the NeuN immunoreactive neurons are remarkably more in the CRUSH+ SPF(I.P.) and CRUSH+ SPF(S.C.)-treated groups compared to the saline-treated group.

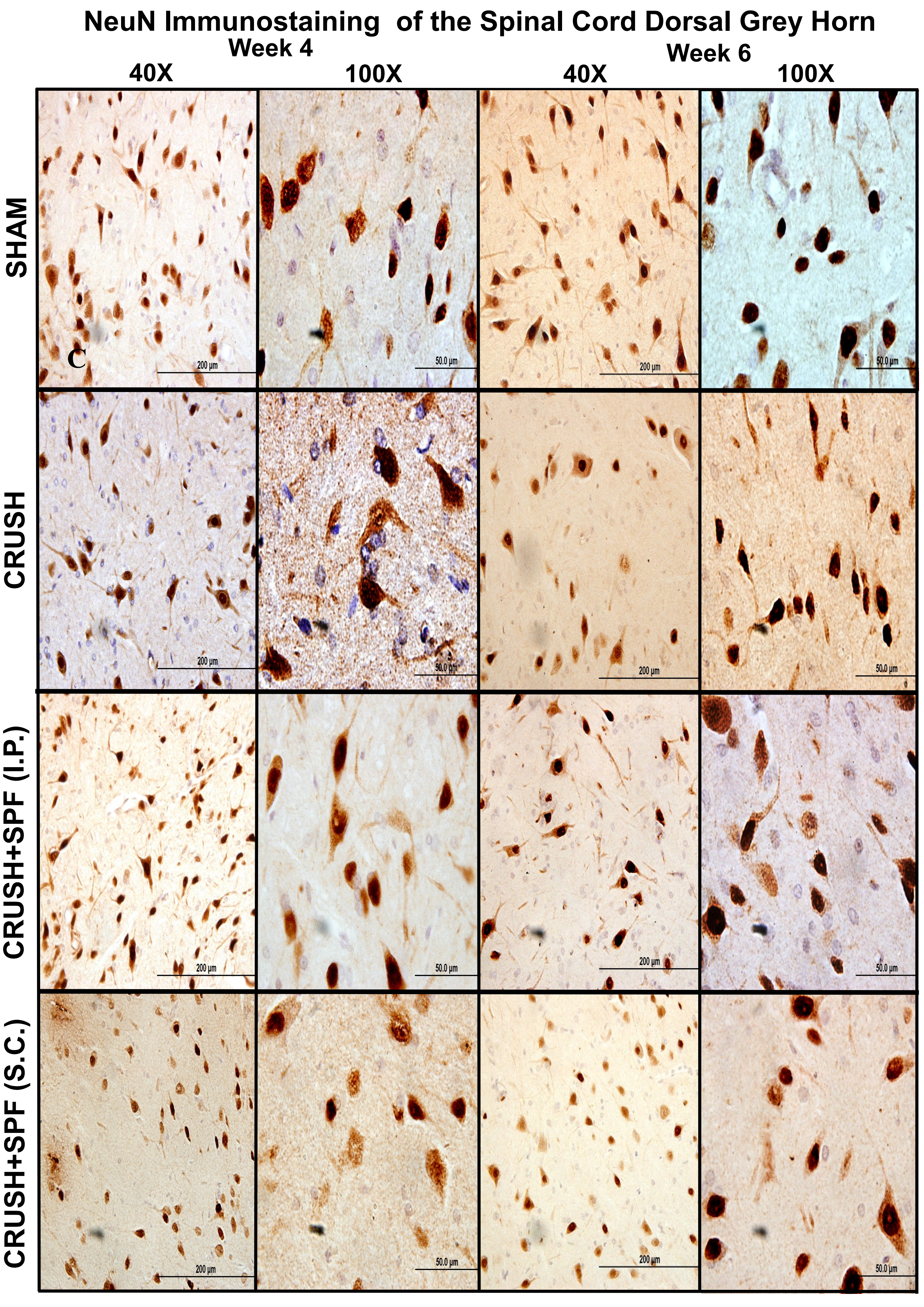
NeuN Immunostaining of the Spinal Cord Dorsal Grey Horn
Week 4
Week 6
40X
100X
40X
100X
SHAM
CRUSH
CRUSH+SPF (I.P.)
CRUSH+SPF (S.C.)
C
200 µm
50.0 µm

Suppl Fig. S6 Representative 10x photomicrographs of the lumbar spinal cord from rats of all groups immunostained for GFAP at Week 4 and Week 6 following nerve crush injury. Note the increasing number of GFAP-immunoreactive astrocytes in the CRUSH rats compared to the SHAM group. The number of GFAP-immunoreactive astrocytes is remarkably less in the CRUSH+0.5X SPF(I.P.) and CRUSH+1X SPF(S.C.)-treated groups compared to the saline-treated group.

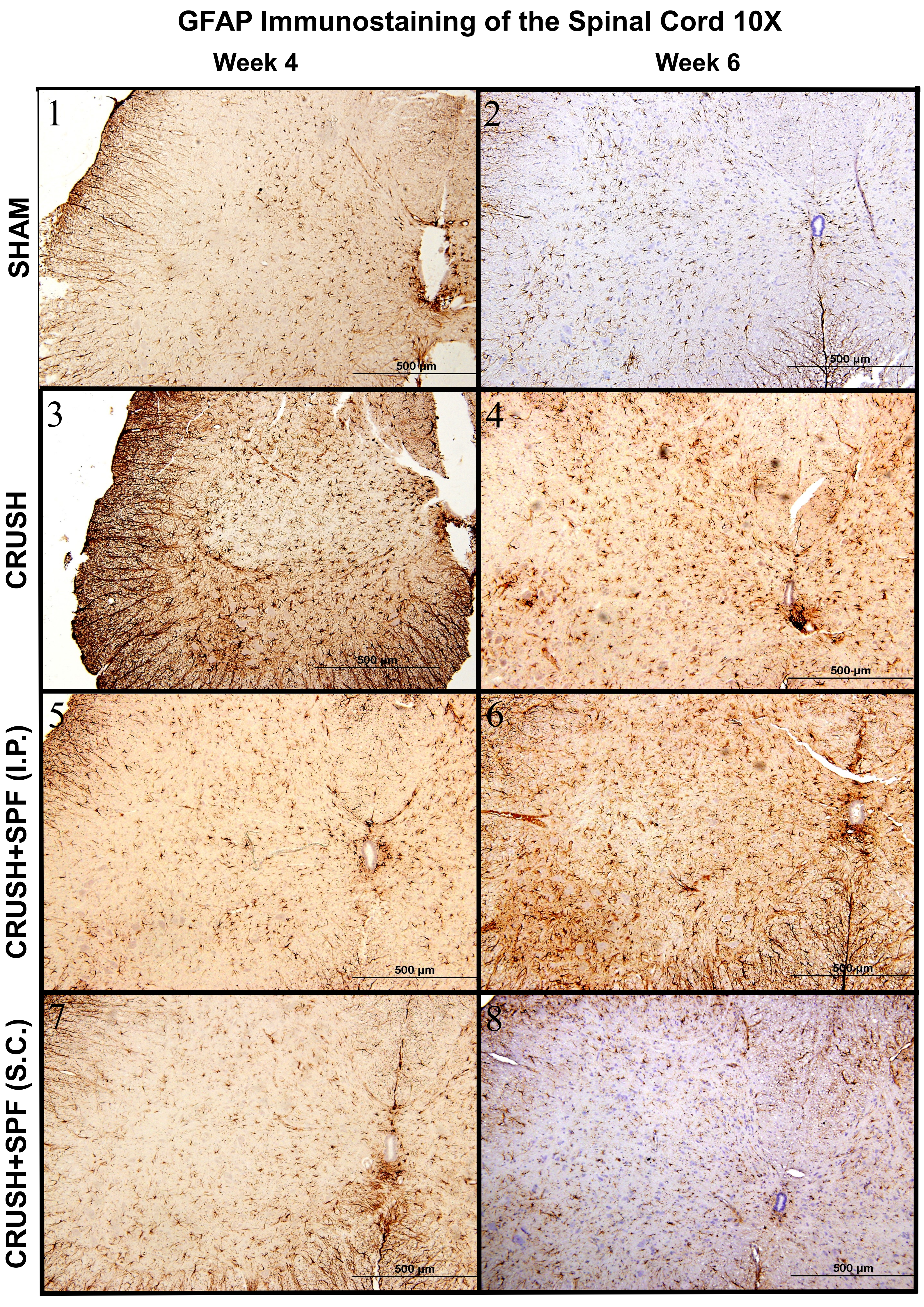
GFAP Immunostaining of the Spinal Cord 10X
Week 4
Week 6
SHAM
CRUSH
CRUSH+SPF (I.P.)
CRUSH+SPF (S.C.)
1
2
3
4
5
6
7
8
500 µm

Suppl. Fig. S7 Representative 40x and 100x photomicrographs of lumbar spinal cord dorsal grey horn from rats of the experimental groups immunostained for NeuN at week 4 and week 6 post-injury. Note less number of neurons and degenerating neurons (arrows) in the sciatic nerve-injured group (CRUSH+SALINE) compared to NAÏVE and SHAM groups. The number of the GFAP immunoreactive neurons are remarkably more in the CRUSH+SPF(I.P.) and CRUSH+SPF(S.C.)-treated groups compared to the saline-treated group.

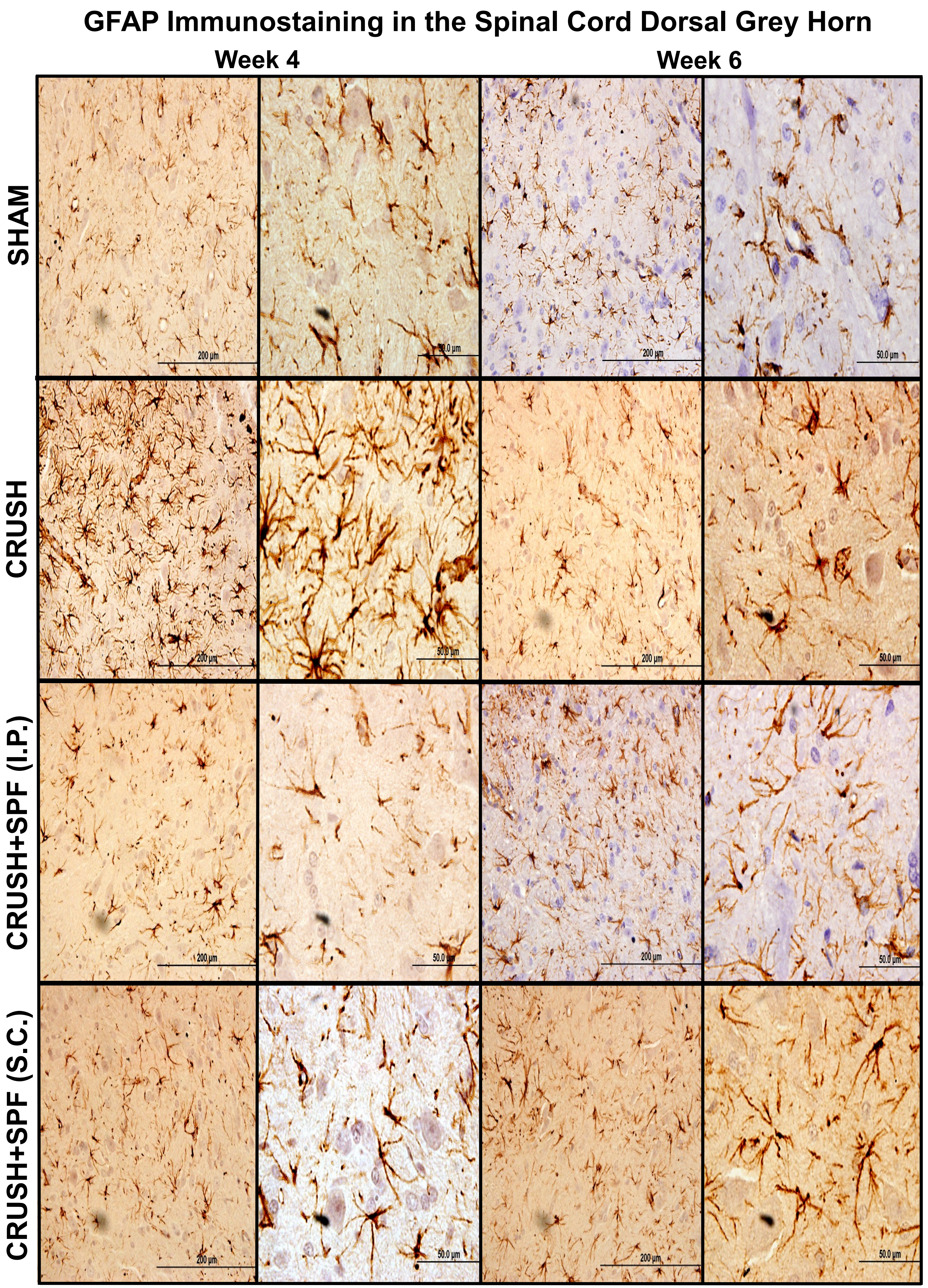
GFAP Immunostaining in the Spinal Cord Dorsal Grey Horn
Week 4
Week 6
SHAM
CRUSH
CRUSH+SPF (I.P.)
CRUSH+SPF (S.C.)
200 µm
50.0 µm

Suppl Fig. S8 Representative 10x photomicrographs of the lumbar spinal cord from rats of all groups immunostained for GAP-43 at week 4 and week 6 following nerve crush injury. Note the increase in the general GAP-43 immunostaining in the CRUSH, CRUSH+0.05X SPF(I.P.) and CRUSH+0.1X SPF(S.C.) compared to SHAM groups at Week 4 following nerve injury. The CRUSH+SPF(I.P.) and CRUSH+SPF(S.C.)-treated groups show a noticeable decrease in the GAP -43 immunoreactivity staining compared to the saline-treated group.

GAP-43 Immunostaining of the Spinal Cord 10X

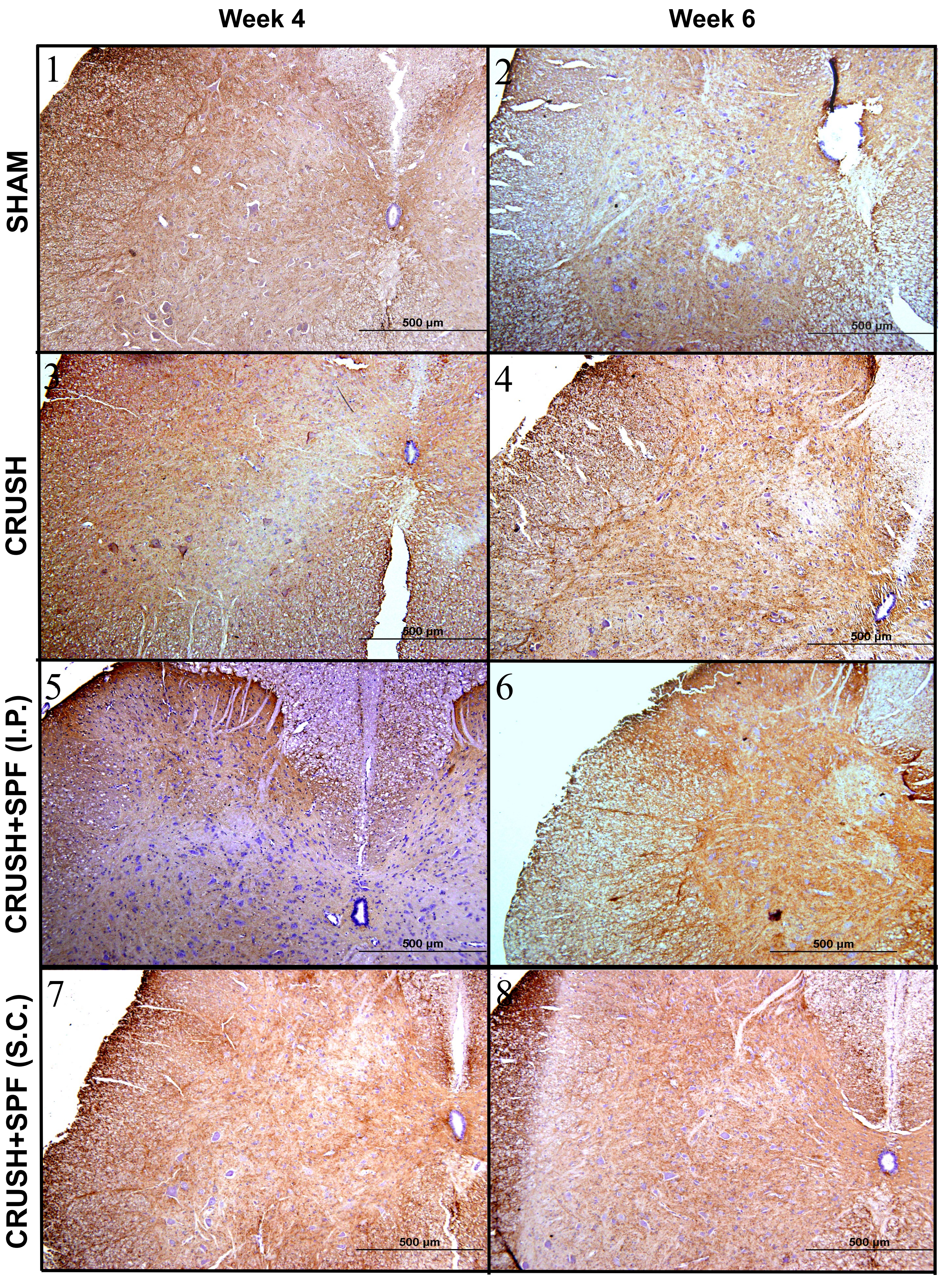

Suppl Fig. S9 Representative 40x and 100x photomicrographs of lumbar spinal cord dorsal grey horn from rats of the experimental groups immune-stained for GAP-43 protein at week 4 and week 6 post-injury. Note the increase in the intensity of GAP-43 immunoreactivity in the sciatic nerve-injured group (CRUSH+SALINE) compared to SHAM groups. The number of the GAP-43 immunoreactivity is remarkably less in the CRUSH+SPF(I.P.) CRUSH+SPF(S.C.)-treated groups compared to the saline-treated group.

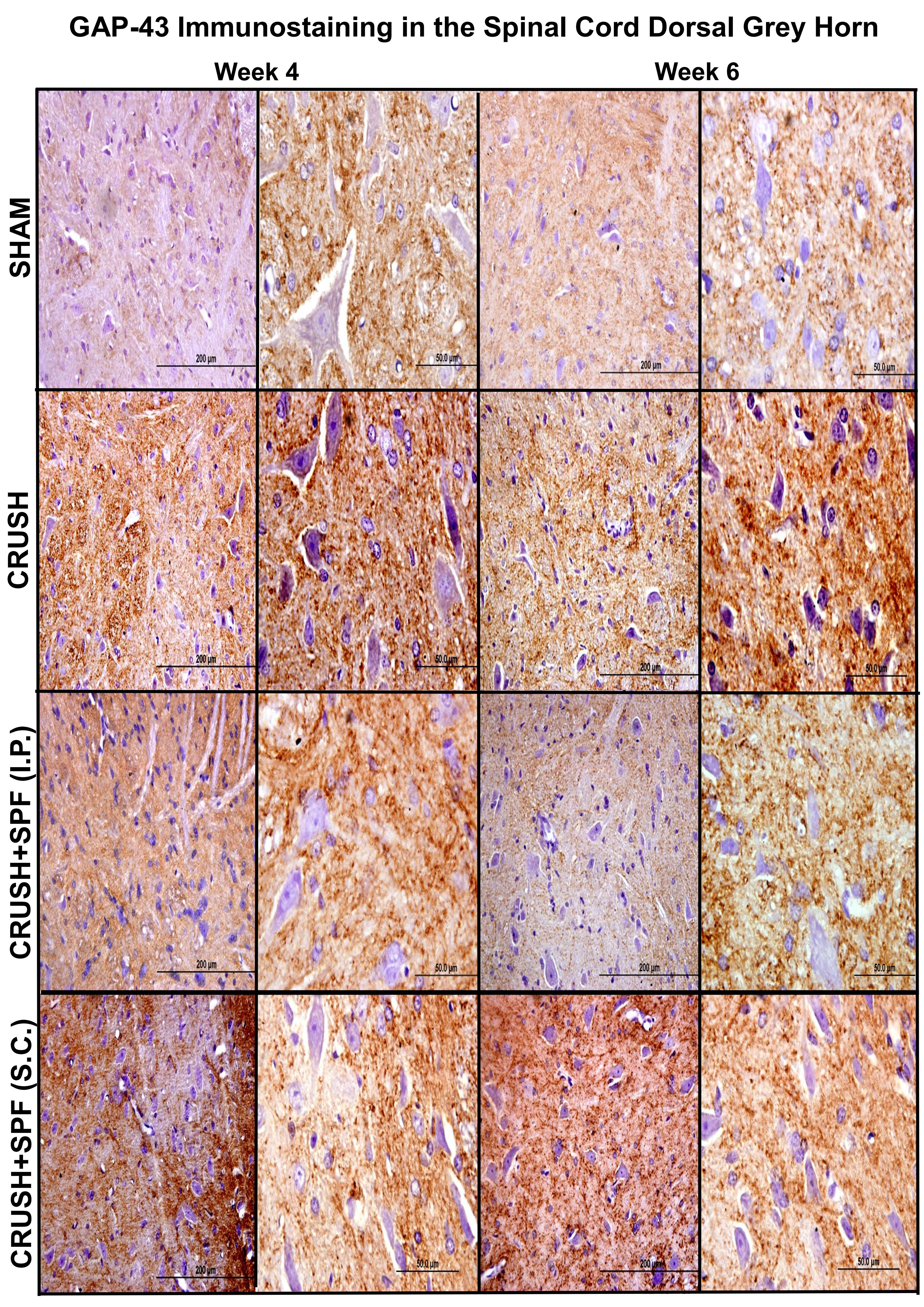

GAP-43 Immunostaining in the Spinal Cord Dorsal Grey Horn
Week 4
Week 6
SHAM
CRUSH
CRUSH+SPF (I.P.)
CRUSH+SPF (S.C.)
200 µm
50.0 µm
